# Supplementary material for: Leucine Signals to mTORC1 via Its Metabolite Acetyl-Coenzyme A
Source: Cell Metab. 2019 Jan 8;29(1):192–201.e7. doi: 10.1016/j.cmet.2018.08.013 (PMC6331339; doi:10.1016/j.cmet.2018.08.013)
Supplement: Document S1. Figures S1–S4 and Table S1 [file mmc1.pdf]

Cell Metabolism, Volume *29*

Supplemental Information

Leucine Signals to mTORC1

via Its Metabolite Acetyl-Coenzyme A

Sung Min Son, So Jung Park, Huikyong Lee, Farah Siddiqi, Jong Eun Lee, Fiona M. Menzies, and David C. Rubinsztein

Figure S1.

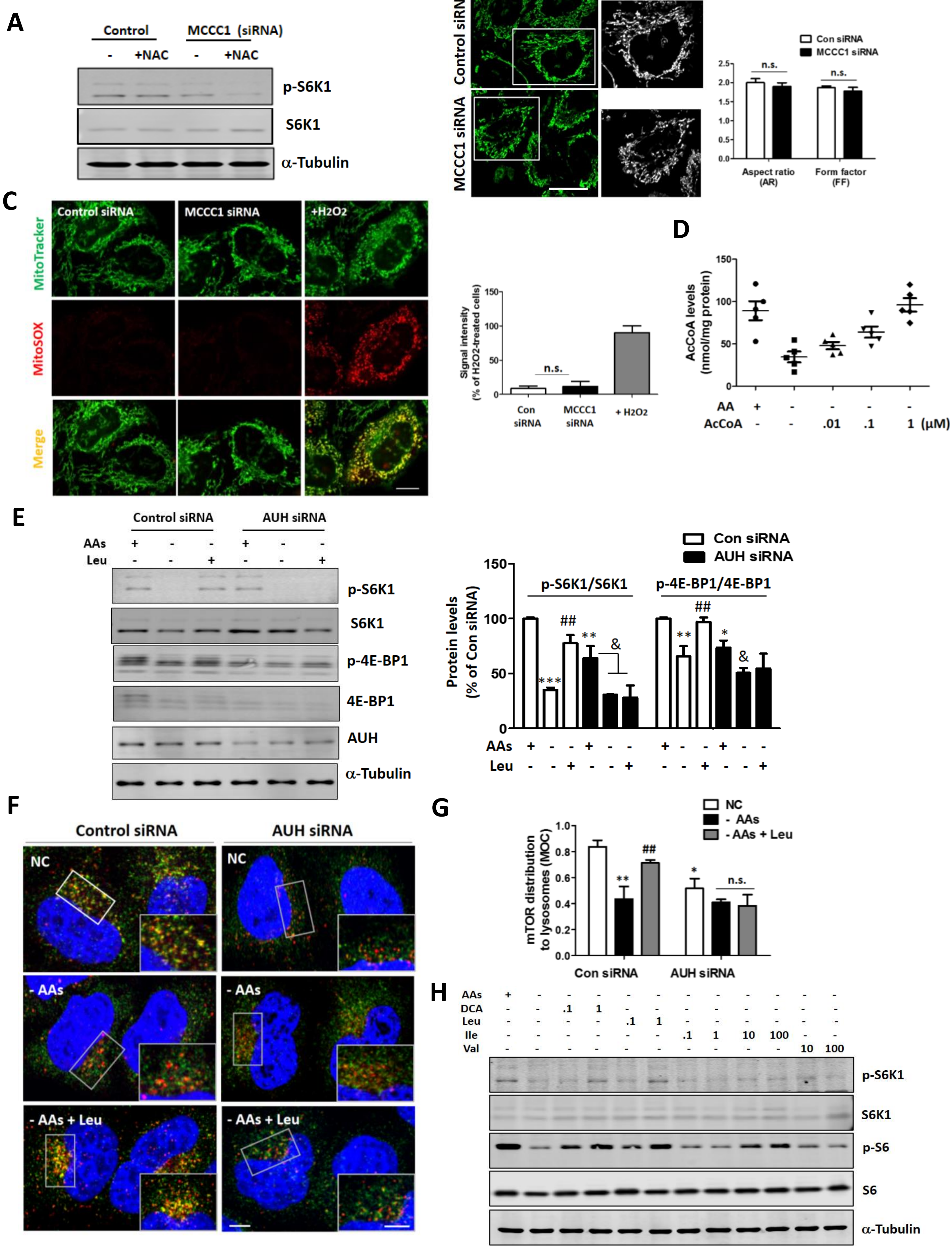
A
Control
MCCC1 (siRNA)
- +NAC - +NAC
p-S6K1
S6K1
α-Tubulin
B
Control siRNA
MCCC1 siRNA
Con siRNA
MCCC1 siRNA
n.s.
n.s.
Aspect ratio (AR)
Form factor (FF)
C
Control siRNA
MCCC1 siRNA
+H2O2
MitoTracker
MitoSOX
Merge
Signal intensity (% of H2O2-treated cells)
n.s.
Con siRNA
MCCC1 siRNA
+ H2O2
D
AcCoA levels (nmol/mg protein)
AA + - - - -
AcCoA - - .01 .1 1 (μM)
E
Control siRNA
AUH siRNA
AAs + - - + - -
Leu - - + - - +
p-S6K1
S6K1
p-4E-BP1
4E-BP1
AUH
α-Tubulin
Con siRNA
AUH siRNA
p-S6K1/S6K1
p-4E-BP1/4E-BP1
Protein levels (% of Con siRNA)
AAs + - - + - - + - - + - -
Leu - - + - - + - - + - - +
F
Control siRNA
AUH siRNA
NC
- AAs
- AAs + Leu
G
NC
- AAs
- AAs + Leu
mTOR distribution to lysosomes (MOC)
n.s.
Con siRNA
AUH siRNA
H
AAs + - - - - - - - - - - -
DCA - - .1 1 - - - - - - - -
Leu - - - - .1 1 - - - - - -
Ile - - - - - - .1 1 10 100 - -
Val - - - - - - - - - - 10 100
p-S6K1
S6K1
p-S6
S6
α-Tubulin

Figure S1. Related to Figure 1.

(A) The role of ROS scavenger N-acetylcysteine (NAC) on mTORC1 regulation in control and MCCC1 knockdown HeLa cells. Blots are representative of at least 3 independent experiments (N=3). p-S6K1 (Thr389).

(B) Mitochondrial morphology in control and MCCC1 knockdown HeLa cells using MitoTracker green dye. Scale bar, 5 μm. Values are mean ± S.E.M. n = about 150 cells. n.s, not significant.

(C) Mitochondrial superoxide levels in control and MCCC1 knockdown HeLa cells. H_2O_2 is a positive control for generation of mitochondrial superoxide. Scale bar, 5 μm. Values are mean ± S.E.M. n = about 100 cells.

(D) AcCoA levels in HeLa cell lysates after AcCoA treatment in the media. We used AcCoA at concentration of 1 μM for this study unless otherwise indicated. N=4.

(E) mTORC1 regulation by Leu under AA starved condition in control and AUH knockdown HeLa cells. * $p < 0.05$, ** $p < 0.01$, *** $p < 0.001$ vs. control cells; ## $p < 0.01$ vs. AA starved cells; & $p < 0.05$ vs. AUH knockdown HeLa cells (two-tailed t-test). NC; normal control. N=3. p-S6K1 (Thr389), p-4E-BP1 (Thr37/46).

(F) Control and AUH knockdown HeLa cells were either left untreated, AA starved for 2 h, or AA starved and then Leu was added for 0.5 h, then immunostained with mTOR (green) and LAMP1 (red) antibodies as shown. Enlarged panels show an overlap between mTOR and LAMP1 signals. The fraction of mTOR-positive lysosomes were determined using Volocity software. Scale bar, 5 μm, 1 μm (enlarged images). NC, normal control.

(G) Quantification data of Figure S1F. Values are mean ± S.E.M. n = about 60 cells. * $p < 0.05$, ** $p < 0.01$ vs. control cells; ## $p < 0.01$ vs. AA starved cells (two-tailed t-test).

(H) mTORC1 regulation by DCA or BCAAs (Leu, Ile and Val). N=3. Unit, μM. p-S6 (Ser235/236).

Figure S2.

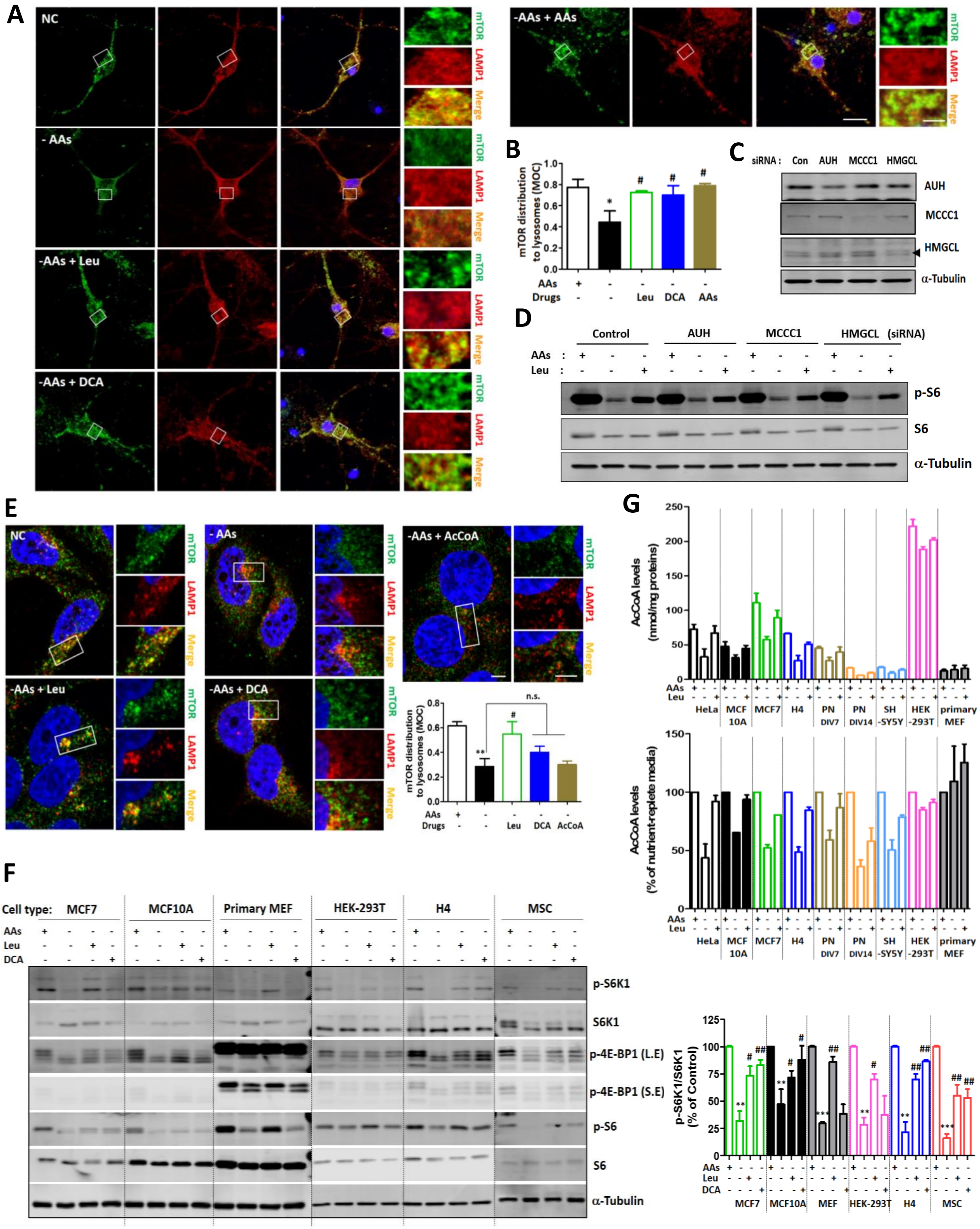
A
NC
- AAs
-AAs + Leu
-AAs + DCA
-AAs + AAs
mTOR
LAMP1
Merge
B
mTOR distribution to lysosomes (MOC)
AAs
Drugs
Leu
DCA
AAs
C
siRNA :
Con
AUH
MCCC1
HMGCL
AUH
MCCC1
HMGCL
α-Tubulin
D
Control
AUH
MCCC1
HMGCL
(siRNA)
AAs :
Leu :
p-S6
S6
α-Tubulin
E
NC
- AAs
-AAs + AcCoA
-AAs + Leu
-AAs + DCA
mTOR
LAMP1
Merge
n.s.
mTOR distribution to lysosomes (MOC)
AAs
Drugs
Leu
DCA
AcCoA
F
Cell type:
MCF7
MCF10A
Primary MEF
HEK-293T
H4
MSC
AAs
Leu
DCA
p-S6K1
S6K1
p-4E-BP1 (L.E)
p-4E-BP1 (S.E)
p-S6
S6
α-Tubulin
p-S6K1/S6K1 (% of Control)
MCF7
MCF10A
MEF
HEK-293T
H4
MSC
G
AcCoA levels (nmol/mg proteins)
AcCoA levels (% of nutrient-replete media)
AAs
Leu
HeLa
MCF 10A
MCF7
H4
PN DIV7
PN DIV14
SH -SY5Y
HEK -293T
primary MEF

Figure S2. Related to Figure 2.

(A) mTORC1 distribution onto lysosomal membrane under AA starvation with or without Leu or DCA in primary neurons. Scale bar, 10 μm, 1 μm (enlarged images). N=4.

(B) Quantification data of Figure S2A. Values are mean ± S.E.M. n = about 40 cells. * $p < 0.05$ vs. control cells; # $p < 0.05$ vs. starved cells (two-tailed t-test).

(C) Efficiency check of siRNAs for AUH, MCCC1 and HMGCL in HEK-293T cells. N=3.

(D) mTORC1 regulation by Leu in the presence or absence of AA, the latter with/without Leu, in control HEK-293T cells or in MCCC1, AUH or HMGCL knockdown cells. N=3.

(E) mTORC1 distribution onto lysosomal membrane under AA starvation with or without Leu, DCA or AcCoA on HEK-293T cells. Values are mean ± S.E.M. n = about 50 cells. ** $p < 0.01$ vs. control cells; # $p < 0.05$ vs. AA starved cells. Scale bar, 2 μm, 0.5 μm (enlarged images).

(F) mTORC1 regulation by Leu or DCA in several cell types. MSC, mesenchymal stem cells. Values are mean ± S.E.M. ** $p < 0.01$, *** $p < 0.001$ vs. control cells; # $p < 0.05$, ## $p < 0.01$ vs. AA starved cells (two-tailed t-test). N=3.

(G) AcCoA levels in several cell types. N=2. PN, primary neurons; DIV, days in vitro. Both actual concentration of AcCoA and % of nutrient-replete media were represented.

Figure S3.

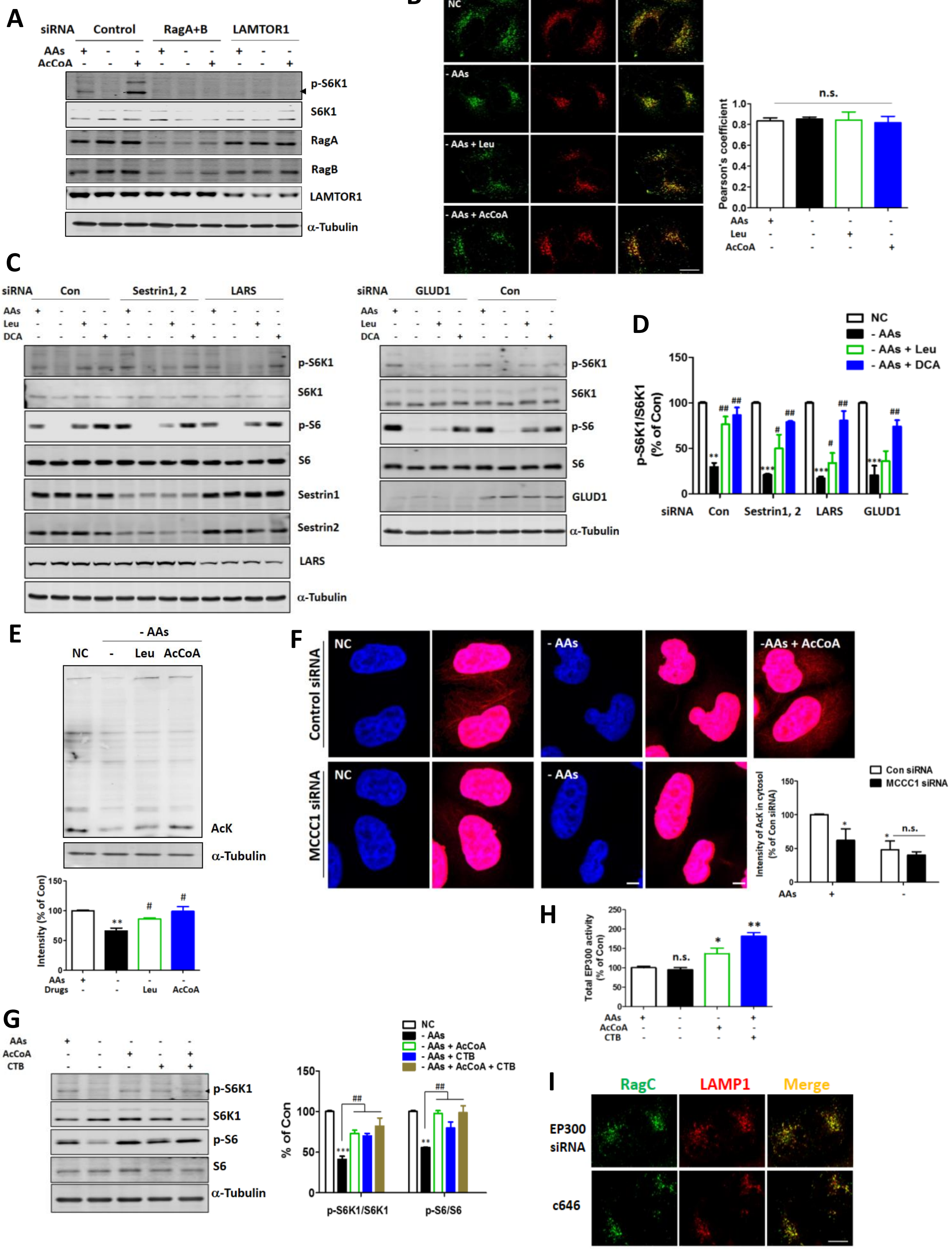
A
siRNA Control RagA+B LAMTOR1
AAs
AcCoA
p-S6K1
S6K1
RagA
RagB
LAMTOR1
α-Tubulin
B
RagC LAMP1 Merge
NC
- AAs
- AAs + Leu
- AAs + AcCoA
n.s.
Pearson's coefficient
AAs
Leu
AcCoA
C
siRNA Con Sestrin1, 2 LARS
siRNA GLUD1 Con
AAs
Leu
DCA
p-S6K1
S6K1
p-S6
S6
Sestrin1
Sestrin2
LARS
α-Tubulin
GLUD1
D
NC
- AAs
- AAs + Leu
- AAs + DCA
p-S6K1/S6K1 (% of Con)
siRNA Con Sestrin1, 2 LARS GLUD1
E
- AAs
NC - Leu AcCoA
AcK
α-Tubulin
Intensity (% of Con)
AAs
Drugs
Leu
AcCoA
F
Control siRNA
MCCC1 siRNA
NC
- AAs
-AAs + AcCoA
Con siRNA
MCCC1 siRNA
Intensity of AcK in cytosol (% of Con siRNA)
n.s.
AAs
G
AAs
AcCoA
CTB
p-S6K1
S6K1
p-S6
S6
α-Tubulin
NC
- AAs
- AAs + AcCoA
- AAs + CTB
- AAs + AcCoA + CTB
% of Con
p-S6K1/S6K1
p-S6/S6
H
Total EP300 activity (% of Con)
n.s.
AAs
AcCoA
CTB
I
RagC LAMP1 Merge
EP300 siRNA
c646

Figure S3. Related to Figure 3.

(A) mTORC1 regulation by AcCoA in control, or RagA/B or LAMTOR1 knockdown HeLa cells. Blots are representative of at least 3 independent experiments. (N=3)

(B) Association of RagC on lysosomal membrane under AA starvation with or without Leu or AcCoA. Values are mean ± S.E.M. n = about 50 cells. Scale bar, 5 μm. n.s., not significant.

(C) mTORC1 regulation by Leu or DCA in Sestrin 1 and 2 (Sestrin1, 2), LARS or GLUD1 knockdown HeLa cells. N=4.

(D) Quantification data of Figure S3C. Values are mean ± S.E.M. ** $p < 0.01$, *** $p < 0.001$ vs. control HeLa cells; # $p < 0.05$, ## $p < 0.01$ vs. AA starved HeLa cells (two-tailed t-test).

(E) Change in global acetylation under AA starved conditions in HeLa cells. Representative immunoblots of total cell lysates showing a change in protein acetylation after 2 h of AA starvation, and then with or without adding with Leu or AcCoA for 0.5 h. ** $p < 0.01$ vs. control cells; # $p < 0.05$ vs. AA starved cells. N=3.

(F) Representative immunofluorescence microphotographs reflecting detection of lysine-acetylated proteins after 2 h of AA starvation with or without adding with AcCoA on control and MCCC1-knockdown HeLa cells. n = about 50 cells. Scale bar represents 2 μm. * $p < 0.01$ vs. control cells.

(G) EP300 activator CTB can rescue mTORC1 activity in AAs starved HeLa cells. ** $p < 0.01$, *** $p < 0.001$ vs. control cells; ## $p < 0.01$ vs. AA starved cells. N=3.

(H) EP300 activity from total cell lysates after AA starvation with/without AcCoA or CTB. * $p < 0.05$, ** $p < 0.01$ vs. control cells. N=3.

(I) Association of RagC on lysosomal membrane on EP300 knockdown or EP300 inhibitor treated cells. n = about 50 cells. Scale bar, 5 μm.

Figure S4.

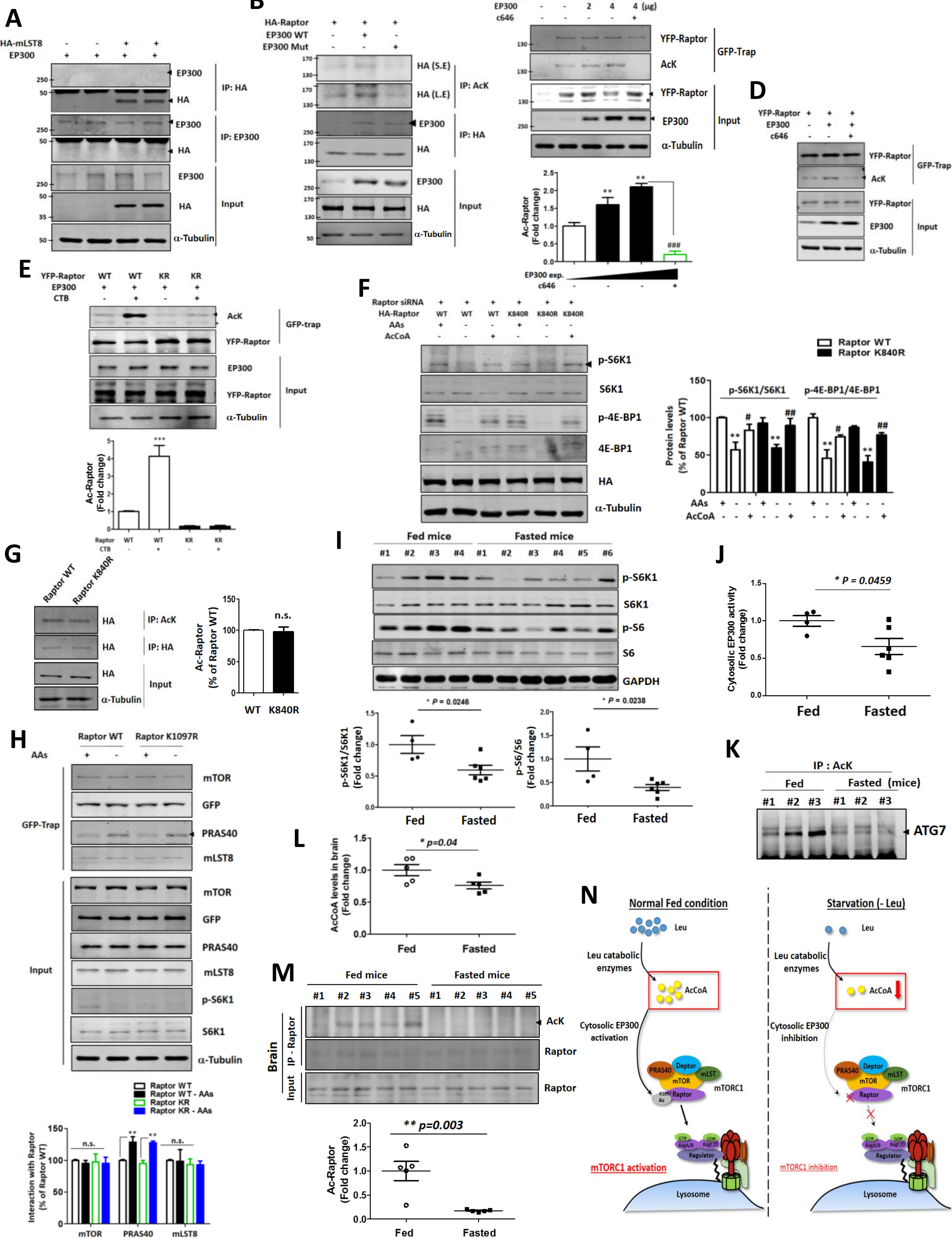
A
HA-mLST8
EP300
IP: HA
IP: EP300
Input
α-Tubulin
B
HA-Raptor
EP300 WT
EP300 Mut
HA (S.E)
HA (L.E)
IP: AcK
IP: HA
C
YFP-Raptor
EP300
c646
GFP-Trap
AcK
Input
Ac-Raptor (Fold change)
EP300 exp.
D
E
CTB
GFP-trap
F
Raptor siRNA
AAs
AcCoA
p-S6K1
S6K1
p-4E-BP1
4E-BP1
Raptor WT
Raptor K840R
p-S6K1/S6K1
p-4E-BP1/4E-BP1
Protein levels (% of Raptor WT)
G
Ac-Raptor (% of Raptor WT)
n.s.
H
Raptor WT
Raptor K1097R
mTOR
GFP
PRAS40
mLST8
Raptor WT - AAs
Raptor KR
Raptor KR - AAs
Interaction with Raptor (% of Raptor WT)
I
Fed mice
Fasted mice
p-S6
S6
GAPDH
* P = 0.0246
* P = 0.0238
p-S6K1/S6K1 (Fold change)
p-S6/S6 (Fold change)
Fed
Fasted
J
* P = 0.0459
Cytosolic EP300 activity (Fold change)
K
IP : AcK
Fasted (mice)
ATG7
L
* p=0.04
AcCoA levels in brain (Fold change)
M
Brain
IP - Raptor
Input
Raptor
** p=0.003
N
Normal Fed condition
Starvation (- Leu)
Leu
Leu catabolic enzymes
AcCoA
Cytosolic EP300 activation
Cytosolic EP300 inhibition
Deptor
mTORC1
Ragulator
Lysosome
mTORC1 activation
mTORC1 inhibition

Figure S4. Related to Figure 4.

(A) mLST8 does not interact with EP300 using IP with HA or EP300 antibody. N=3.

(B) Acetylation of Raptor by EP300 acetyltransferase with EP300 WT and catalytically-inactive EP300 construct (EP300 Mut). EP300 can bind Raptor and acetylate it. S.E., short exposure; L.E., long exposure. N=4.

(C and D) Acetylation of Raptor by EP300 in HeLa (C) and HEK-293T cells (D). EP300 inhibitor c646 inhibits acetylation of Raptor. ** $p < 0.01$ vs. YFP-Raptor expressing cells without transfection of exogenous EP300 cDNA; ### $p < 0.001$ vs. YFP-Raptor and exogenous EP300 expressing cells. N=3.

(E) Acetylation of Raptor on Raptor WT or KR expressing HeLa cells with/without CTB. *** $p < 0.001$ vs. YFP-Raptor expressing cells. N=3.

(F) Cells were depleted of Raptor with siRNA and reconstituted with Raptor wild-type (WT) or K840R mutant, then analysed for mTORC1 activity in the presence or absence of AA, the latter with/without AcCoA in HeLa cells. ** $p < 0.01$ vs. control cells; # $p < 0.05$, ## $p < 0.01$ vs. AA starved cells. N=3.

(G) Acetylation of WT and K840R Raptor. K840 site in Raptor is not acetylated in HeLa cells. N=3. n.s., not significant.

(H) Binding of Raptor WT or K1097R mutant with components of mTORC1. ** $p < 0.01$ vs. control cells. N=3.

(I-K) Decreased mTORC1 (I), EP300 activity (J) and ATG7 acetylation (K) in fasted mouse brains. Brain samples from fed (n=4) and fasted mice (n=6) were analysed. * $p < 0.05$ vs. fed mice.

(L and M) Decreased AcCoA levels and acetylation of Raptor in brains of mice fasted for 23 h. The tissue samples from fed (n=5) and fasted mice (n=5) were analysed for AcCoA (L) and acetylated Raptor (M) levels. * $p < 0.05$, ** $p < 0.01$ vs. fed mice.

(N) Schematic diagram of this study. AA starvation induces decreased AcCoA levels both total and cytosolic fraction that promote the translocation of EP300 into nucleus. Because EP300 acetyltransferase can bind Raptor and acetylate it at K1097 site which is important for interaction with Rag complex, AA starvation induces Raptor deacetylation, and then results in inhibition of interaction with Rag complex on the lysosomal membrane. The knockdown of Leu catabolic enzymes (MCCC1 and AUH) shows similar pattern with AA starvation, suggesting Leu metabolite AcCoA has an essential role on sensing Leu for mTORC1

activation. Interestingly, this mechanism appears to be relevant in a variety of cell types, including HeLa and neurons, not MEF and HEK-293T cells.

Table S1. siRNA sequences, Related to STAR Methods

Non-targeting SMARTpool siRNA<br>UGGUUUACAUGUCGACUAA<br>UGGUUUACAUGUUGUGUGA<br>UGGUUUACAUGUUUUCUGA<br>UGGUUUACAUGUUUUCCUA	Dharmacon	D-001810-10
RagA siRNAa: CCGAUGAAGUUCUGCUGUU	Dharmacon	J-016070-05
RagA siRNAb: GACAAUAUCUUCCGUAACG	Dharmacon	J-016070-06
RagA siRNAc: GAACUCUCCUGACGCCAAA	Dharmacon	J-016070-07
RagA siRNAd: UCGAUAAUCUUCGCCAAUU	Dharmacon	J-016070-08
RagB siRNAa: GAAGAAGAUUUGAGGCGUU	Dharmacon	J-012189-09
RagB siRNAb: GGGACAACAUCUUCCGAAA	Dharmacon	J-012189-10
RagB siRNAc: AGUGUAAAGAGCAGCGUGA	Dharmacon	J-012189-11
RagB siRNAd: CUGAGAAAACGACGGAGAA	Dharmacon	J-012189-12
LAMTOR1 siRNAa: GUUUGUCACCCUCGAUAAA	Dharmacon	J-020916-17
LAMTOR1 siRNAb: AAGUGAGGGUAGAACCUUU	Dharmacon	J-020916-18
LAMTOR1 siRNAc: GGCUUAUACAGUACCCUAA	Dharmacon	J-020916-19
LAMTOR1 siRNAd: UCUCCAGGAUAGCUGCUUA	Dharmacon	J-020916-20
AUH siRNAa: GAGCAGUGAUUAACGAUAU	Dharmacon	J-008457-05
AUH siRNAb: GGAGGUCGAUUUAGUAACA	Dharmacon	J-008457-06
AUH siRNAc: AAAGUACGGACCAUAAUAA	Dharmacon	J-008457-07
AUH siRNAd: GAAGAAGCUUGUUAUGCUC	Dharmacon	J-008457-08
HMGCL siRNAa: AAGCAGGACUCUCUGUUAU	Dharmacon	J-019290-05
HMGCL siRNAb: CGUAUCUACUCCAGUGAAA	Dharmacon	J-019290-06
HMGCL siRNAc: UCAAUGGGCUGCUACGAGA	Dharmacon	J-019290-07
HMGCL siRNAd: UCUGAAAGCUGGAAACUUU	Dharmacon	J-019290-08
KAT2A siRNAa: AGGACAAAUUGGUGCCCGA	Dharmacon	J-008457-17
KAT2A siRNAb: GUUCCUGGCAUUCGAGAGA	Dharmacon	J-008457-18
KAT2A siRNAc: GCUACUACGUGACCCGGAA	Dharmacon	J-008457-19
KAT2A siRNAd: ACUCAUGUCUUUGGGCGAA	Dharmacon	J-008457-20
KAT2B siRNAa: GGUACUACGUGUCUAAGAA	Dharmacon	J-005055-05
KAT2B siRNAb: GAGCCGACCUGCAGCAAAU	Dharmacon	J-005055-06
KAT2B siRNAc: CGACAGAUUCCUAUAGAAA	Dharmacon	J-005055-07
KAT2B siRNAd: GCAAACAAUAGUUGAGUUG	Dharmacon	J-005055-08
KAT5 siRNAa: GCGUCCAUUACAUUGACUU	Dharmacon	J-006301-05
KAT5 siRNAb: GCCUCAAUCUCAUCAACUA	Dharmacon	J-006301-06
KAT5 siRNAc: CUAGGUACCUAGCUUCGAU	Dharmacon	J-006301-07
KAT5 siRNAd: GAUGGUACCAUCAGUGCCC	Dharmacon	J-006301-08
KAT8 siRNAa: GGGAACUACGAAAUUGGGU	Dharmacon	J-014800-05
KAT8 siRNAb: AUCCCUCAAUAGGUACCUA	Dharmacon	J-014800-06
KAT8 siRNAc: CUACGUGGGCUUUAAGGUA	Dharmacon	J-014800-07

KAT8 siRNAd: AUGCCUGGUAUUUGGUACC	Dharmacon	J-014800-08
EP300 siRNAa: GGACUACCCUAUCAAGUAA	Dharmacon	J-003486-11
EP300 siRNAb: GACAAGGGAUAAUGCCUAA	Dharmacon	J-003486-12
EP300 siRNAc: GUUCAAUAAUGCCUGGUUA	Dharmacon	J-003486-13
EP300 siRNAd: CGACAGGGAUGCAGCAACA	Dharmacon	J-003486-14
Raptor siRNAa: UGGCUAGUCUGUUUCGAAA	Dharmacon	J-004107-05
Raptor siRNAb: CACGGAAGAUGUUCGACAA	Dharmacon	J-004107-06
Raptor siRNAc: AGAAGGGCAUUACGAGAUU	Dharmacon	J-004107-07
Raptor siRNAd: UGGAGAAGCGUGUCAGAUA	Dharmacon	J-004107-08
Sestrin1 siRNAa: GAGGAGGAUCAGCGGUUGA	Dharmacon	J-020244-05
Sestrin1 siRNAb: GCUCAAUGGUUUAGAGAAU	Dharmacon	J-020244-06
Sestrin1 siRNAc: CAGCAGAGAUUCAACUACU	Dharmacon	J-020244-07
Sestrin1 siRNAd: UUAGUGAACCUGCAUGUAA	Dharmacon	J-020244-08
Sestrin2 siRNAa: GGAGGGAGUAUUAGAUUAU	Dharmacon	J-019134-17
Sestrin2 siRNAb: GCAGGGACCCGUUGAACAA	Dharmacon	J-019134-18
Sestrin2 siRNAc: GCGCAAACUCAGCGAGAUC	Dharmacon	J-019134-19
Sestrin2 siRNAd: CUUUCGGAUAUGAGGACUU	Dharmacon	J-019134-20
LARS siRNAa: GCAAAGAACUAUCCACCUU	Dharmacon	J-010171-05
LARS siRNAb: GGGAAGCGGUAUACAAUUU	Dharmacon	J-010171-06
LARS siRNAc: UAAGUGGCCUGAAAGGUAA	Dharmacon	J-010171-07
LARS siRNAd: GAAAUAGAGCUGUAUGGUU	Dharmacon	J-010171-08
ACLY siRNAa: GCACGAAGUCACAAUCUUU	Dharmacon	J-004915-05
ACLY siRNAb: CGAGUGAAGUCGAUAAACA	Dharmacon	J-004915-06
ACLY siRNAc: GAGAGCAAUUCGAGAUUAC	Dharmacon	J-004915-07
ACLY siRNAd: CCACUCCUCUGCUCGAUUA	Dharmacon	J-004915-08
MCCC1 siRNAa: CCAUGAAGUACACAACAGC	Ambion	AM51331
MCCC1 siRNAb: CCAAGUCCUUGGUAAUCUU	Ambion	s32399
MCCC1 siRNAc: GAUCCUAGCAAUAACUUCA	Ambion	s32400
MCCC1 siRNAd: GAGUUGCUAGUAAAGCGAA	Ambion	s32401
GLUD1 siRNA: GGGCCUUUUGAACAGUAGU	Ambion	AM16708
