# Supplementary material for: Leucine Signals to mTORC1 via Its Metabolite Acetyl-Coenzyme A
Source: Cell Metab. 2019 Jan 8;29(1):192–201.e7. doi: 10.1016/j.cmet.2018.08.013 (PMC6331339; doi:10.1016/j.cmet.2018.08.013)
Supplement: Document S2. Article plus Supplemental Information [file mmc2.pdf]

Short Article

Cell Metabolism

Leucine Signals to mTORC1 via Its Metabolite Acetyl-Coenzyme A

Graphical Abstract

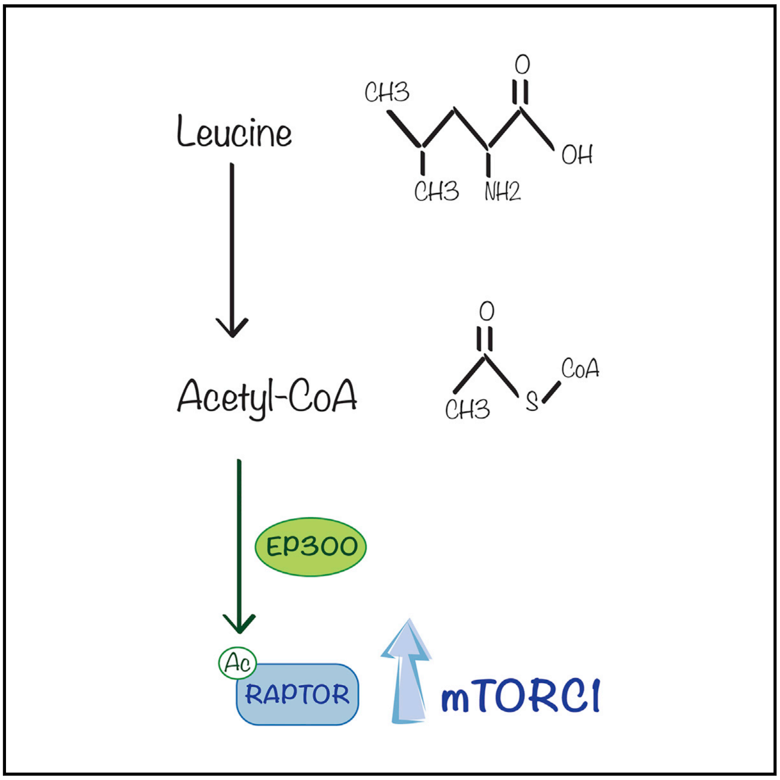

Highlights

- Leucine metabolizing enzymes impact mTORC1 activity
- AcCoA, the final leucine metabolite, regulates mTORC1 activity by Raptor acetylation
- AcCoA regulates mTORC1 in a cell-type-specific manner
- Fasted tissues in mice have decreased AcCoA, acetylated Raptor, and mTORC1 activity

Authors

Sung Min Son, So Jung Park, Huikyong Lee, Farah Siddiqi, Jong Eun Lee, Fiona M. Menzies, David C. Rubinsztein

Correspondence

dcr1000@cam.ac.uk

In Brief

Son et al. find that the leucine metabolite, acetyl-coenzyme A, promotes mTORC1 activity through EP300-mediated acetylation of Raptor, an mTORC1 regulator. The pathway that is cell-type specific, allows for intermediary enzymes to regulate mTORC1 activity, without a need for leucine sensing. In fasted mice, Raptor acetylation decreases in some tissues, leading to mTORC1 inhibition.

Son et al., 2019, Cell Metabolism *29*, 192–201
January 8, 2019 © 2018 The Author(s). Published by Elsevier Inc.
https://doi.org/10.1016/j.cmet.2018.08.013

Cell Metabolism
Short Article

Leucine Signals to mTORC1 via Its Metabolite Acetyl-Coenzyme A

Sung Min Son,[1,2] So Jung Park,[1] Huikyong Lee,[1] Farah Siddiqi,[1] Jong Eun Lee,[3] Fiona M. Menzies,[1] and David C. Rubinsztein[1,2,4,*]
[1]Department of Medical Genetics, Cambridge Institute for Medical Research (CIMR), University of Cambridge, Cambridge, UK
[2]UK Dementia Research Institute, Cambridge Institute for Medical Research (CIMR), University of Cambridge, Cambridge, UK
[3]Department of Anatomy, Bk21 Plus Project for Medical Sciences and Brain Research Institute, Yonsei University College of Medicine, Seoul 03722, Republic of Korea
[4]Lead Contact
*Correspondence: dcr1000@cam.ac.uk
https://doi.org/10.1016/j.cmet.2018.08.013

SUMMARY

The mechanistic target of rapamycin (mTOR) complex 1 (mTORC1) is a master regulator of cell growth and metabolism. Leucine (Leu) activates mTORC1 and many have tried to identify the mechanisms whereby cells sense Leu in this context. Here we describe that the Leu metabolite acetyl-coenzyme A (AcCoA) positively regulates mTORC1 activity by EP300-mediated acetylation of the mTORC1 regulator, Raptor, at K1097. Leu metabolism and consequent mTORC1 activity are regulated by intermediary enzymes. As AcCoA is a Leu metabolite, this process directly correlates with Leu abundance, and does not require Leu sensing via intermediary proteins, as has been described previously. Importantly, we describe that this pathway regulates mTORC1 in a cell-type-specific manner. Finally, we observed decreased acetylated Raptor, and inhibited mTORC1 and EP300 activity in fasted mice tissues. These results provide a direct mechanism for mTORC1 regulation by Leu metabolism.

INTRODUCTION

The evolutionarily conserved mechanistic target of rapamycin (mTOR) complex 1 (mTORC1) is a master regulator of cell growth and metabolism (Jewell et al., 2013; Laplante and Sabatini, 2012), and comprises mTOR, regulatory-associated protein of mammalian target of rapamycin (Raptor), mTOR-associated protein, LST8 homolog, proline-rich Akt substrate of 40 kDa, and DEP domain-containing mTOR-interacting protein (Laplante and Sabatini, 2012). Dysregulation of mTORC1 is common in human diseases, including cancer, diabetes, and neurodegenerative diseases, such as Alzheimer's disease and Parkinson's disease (Laplante and Sabatini, 2012; Saxton and Sabatini, 2017; Zoncu et al., 2011). In mammals, growth factors and intracellular energy signals stimulate mTORC1 activity through inhibition of the tuberous sclerosis complex (TSC) 1 and 2, a negative regulator of mTORC1 (Dibble and Manning, 2013). Amino acids (AAs) signal to mTORC1 through Rag GTPases independently of the TSC complex (Jewell et al., 2013; Sancak et al., 2008). Previous reports showed that Rag heterodimers interact with mTORC1 and that AAs induce the mTORC1-Rag interaction by promoting the loading of RagB with GTP, enabling it to directly interact with Raptor (Sancak et al., 2008). Activation of the mTORC1 pathway by AAs correlates with the translocation of mTORC1 from undefined locations to lysosomes where it encounters RHEB, a potent mTORC1 activator (Efeyan et al., 2012; Sancak et al., 2008). The Ragulator complex, which is encoded by the *MAPKSP1*, *ROBLD3*, and *c11orf59* genes (Sancak et al., 2010), interacts with the Rag GTPases, recruits them to lysosomes, and is essential for mTORC1 activation (Sancak et al., 2010).

Among AAs, leucine (Leu) has been implicated in mTORC1 activation (Hara et al., 1998; Sancak et al., 2008) and many have searched for the Leu sensor(s) in cells that control mTORC1 activity (Han et al., 2012; Lorin et al., 2013; Saxton et al., 2016; Wolfson et al., 2016; Zheng et al., 2016). Recently, Sestrin2, a GATOR2-interacting protein that inhibits mTORC1 (Chantranupong et al., 2014; Parmigiani et al., 2014; Saxton et al., 2016), was reported as an intracellular Leu sensor for mTORC1 pathway in HEK293T cells (Wolfson et al., 2016). Other proposed Leu sensors include leucyl-tRNA synthetase (LARS) (Han et al., 2012; He et al., 2018) and glutamate dehydrogenase (GLUD1) (Lorin et al., 2013). Here, by studying enzymes regulating the metabolism of Leu to acetyl-coenzyme A (AcCoA), we have discovered that Leu signaling to mTORC1 does not necessarily require a "sensor" in some cell lines and primary cells, as AcCoA positively regulates mTORC1 via Raptor acetylation.

RESULTS AND DISCUSSION

MCCC1, Which Regulates Leu Metabolism, Impacts mTORC1 Signaling in HeLa Cells

To determine whether Leu catabolism can regulate mTORC1 in HeLa cells, we knocked down MCCC1, a key enzyme in the Leu metabolic pathway (Figure 1A) (Chu and Cheng, 2007), which decreased levels of markers of mTORC1 activity: phosphorylated S6K1, 4E-BP1 (mTORC1 kinase substrates), and S6 (S6K1 substrate) (Figure 1B). When *MCCC1* cDNA was transfected into MCCC1 knockdown cells, it rescued mTORC1 activity (Figure 1C). These data suggested that MCCC1 could

 © 2018 The Author(s). Published by Elsevier Inc.
This is an open access article under the CC BY license (http://creativecommons.org/licenses/by/4.0/).

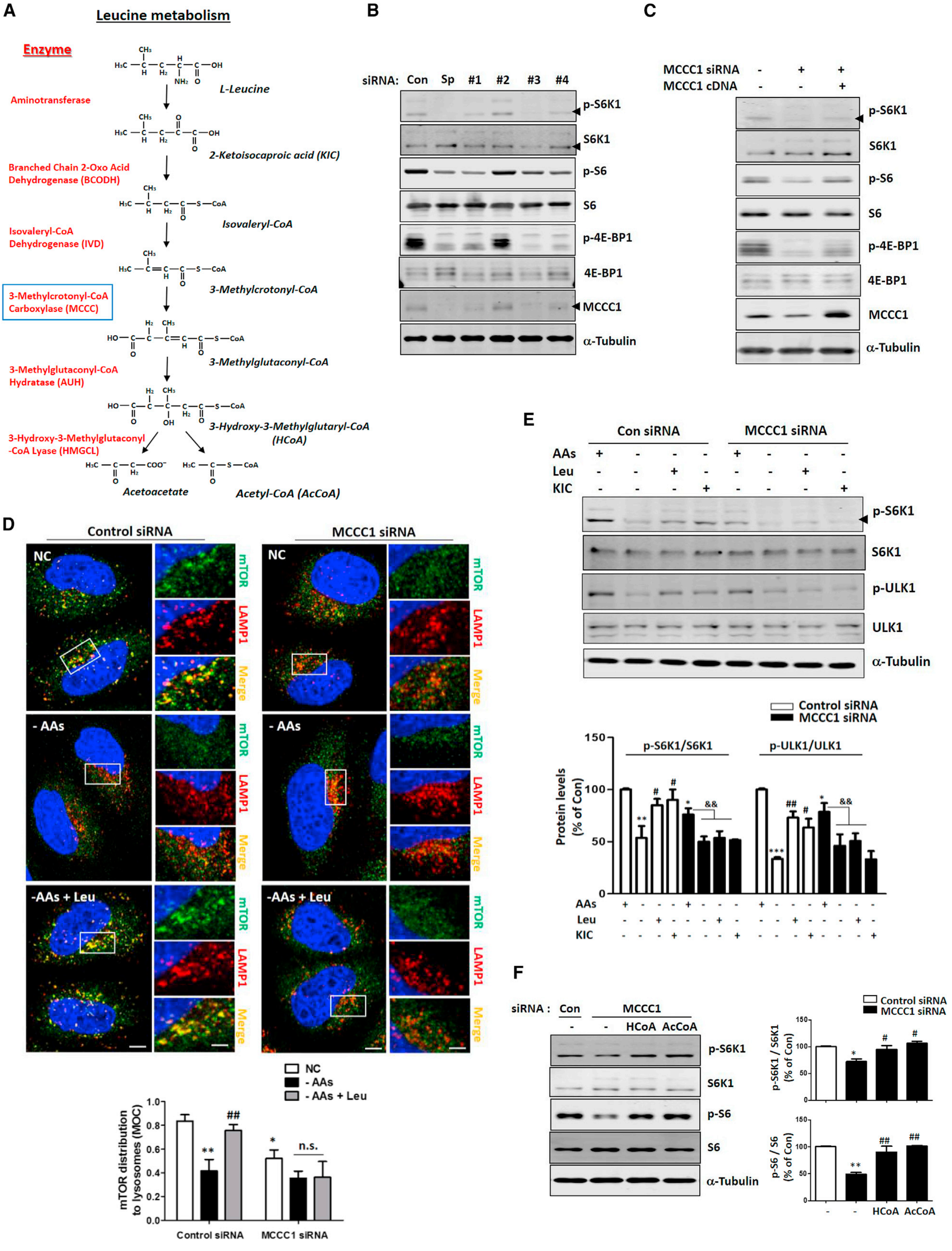

(legend on next page)

regulate mTORC1. MCCC1 knockdown did not obviously perturb mitochondrial morphology or cause any reactive oxygen species (ROS) elevation, and N-acetylcysteine, an ROS scavenger, did not rescue mTORC1 inhibition in MCCC1 knockdown cells (Figures S1A–S1C). Since treatment with Leu stimulates lysosomal recruitment and activation of mTORC1 under AA starvation conditions, we determined whether MCCC1 similarly affected the lysosomal translocation of mTORC1. When we added Leu to AA-starved cells, mTORC1 appeared in puncta-like structures that co-localized with LAMP1-positive vesicles (late endosomes/lysosomes) in control cells (Figure 1D, left panel), but the mTORC1 redistribution onto lysosomes was reduced upon knockdown of MCCC1 (Figure 1D, right panel). Similarly, under AA starvation conditions, neither Leu nor its direct metabolite alpha-ketoisocaproate, which is upstream of MCCC1 (Figure 1A), rescued the mTORC1 pathway in MCCC1 knockdown cells (Figures 1D and 1E). However, 3-hydroxy-3-methylglutaryl-coenzyme A and 1 μM AcCoA (Figure S1D shows that this results in physiologically relevant levels intracellularly), Leu metabolites downstream of MCCC1 (Figure 1A), could restore mTORC1 activity in MCCC1 knockdown cells (Figure 1F), indicating that Leu catabolism is essential for mTORC1 regulation. As we observed with MCCC1 knockdown, depletion of AUH (the enzyme immediately downstream of MCCC1 in the pathway from Leu to AcCoA; Figure 1A) decreased mTORC1 activity, and Leu treatment failed to rescue mTORC1 activity in AA-starved, AUH knockdown cells (Figures S1E–S1G). To determine whether other branched chain AAs can also regulate mTORC1, we treated starved cells with isoleucine (Ile) and valine (Val). Val had no effect, and only high concentrations of Ile could rescue mTORC1 activity in AA-starved cells (Figure S1H).

AcCoA, the Final Leu Metabolite, Regulates mTORC1 under AA-Starved Conditions in Some Cell Types

Total and cytosolic AcCoA levels were decreased in both MCCC1 knockdown HeLa cells and AA-starved HeLa cells (Figures 2A and 2B). Leu and dichloroacetate (DCA, which enhances AcCoA levels by activating pyruvate dehydrogenase to produce AcCoA from pyruvate rather than Leu; Marino et al., 2014) rescued the reduction of AcCoA under AA starvation conditions (Figure 2B). Leu metabolites, including AcCoA and DCA, rescued mTORC1 inhibition by AA starvation (Figures S1H and 2C–2E). To determine whether cytosolic AcCoA levels are important for this mTORC1 rescue in AA-starved HeLa cells, we knocked down ATP citrate lyase (ACLY), which makes a major contribution to cytosolic AcCoA production in many cell types (Marino et al., 2014). In ACLY knockdown HeLa cells, total and cytosolic AcCoA levels were decreased (Figure 2F), and the increase of mTORC1 activity mediated by Leu or DCA in-starved cells was blunted (Figure 2G). Next, as we observed in HeLa cells, Leu and DCA rescued the inhibition of mTORC1 resulting from AA starvation in human neuroblastoma SH-SY5Y cells (Figure 2H). Likewise DCA could rescue mTORC1 activity in Mccc1 knockdown primary neurons (Figure 2I). Furthermore, we found that Leu metabolites rescued mTORC1 inhibition resulting from AA starvation in primary neurons, as we observed in SH-SY5Y or in HeLa cells (Figure 2J), which we confirmed by immunostaining with an anti-phosphorylated S6 antibody (Figure 2K). Consistent with these data, mTORC1 redistribution onto lysosomes in primary neurons was reduced under AA starvation conditions, and this was rescued by the addition of Leu or DCA (Figures S2A and S2B). Interestingly, unlike HeLa and neuronal cells, HEK293T cells showed different patterns of mTORC1 regulation (Figures S2C–S2E), which did not appear to be obviously dependent on Leu metabolism. We found that DCA could reverse mTORC1 inhibition by AA starvation in epithelial cell lines (MCF7 and MCF10A), a glial cell line (H4), and mesenchymal stem cells, but not in primary mouse embryonic fibroblasts (MEFs) and HEK293T cells (Figure S2F). We analyzed AcCoA levels in cell lines where mTORC1 signaling after starvation was sensitive/insensitive to DCA/AcCoA. Our data suggest that non-responsiveness may occur either when cells have very high AcCoA levels in nutrient replete media, or if they fail to lower AcCoA levels after AA starvation (Figure S2G).

Next, we investigated the mechanism by which Leu metabolites regulate mTORC1. Since Rag GTPase recruits mTORC1 to lysosomes (Jewell et al., 2013) and AA starvation with/without Leu metabolites regulates mTORC1 lysosomal localization (Figures 1D and 2D), we assessed if the Ragulator complex on lysosomes influenced mTORC1 regulation by AcCoA. RagA and RagB double knockdown or knockdown of LAMTOR1 (which anchors the Ragulator complex to lysosomes) abrogated the ability of AcCoA to rescue mTORC1 inhibition in AA-starved HeLa cells (Figure S3A). Addition of Leu or AcCoA to starved cells did not alter the localization of the Rag complex on lysosomes (Figure S3B), indicating that Leu metabolites can regulate mTORC1

Figure 1. MCCC1, Which Regulates Leu Metabolism, Modifies mTORC1 Signaling in HeLa Cells

(A) Leu metabolic pathway. Blue box shows MCCC1 protein.

(B) Control and MCCC1 knockdown (transfected with pool or four deconvoluted oligos) HeLa cells were used to determine whether MCCC1 can regulate mTORC1 signal. Blots are representative of at least three independent experiments (N = 3). P- indicates phosphorylated protein. Note that oligo no. 2 has not knocked down MCCC1. p-S6K1 (Thr389), p-S6 (Ser235/236), p-4E-BP1 (Thr37/46).

(C) Re-introduction to MCCC1 knockdown HeLa cells with MCCC1 cDNA. Blots are representative of at least three independent experiments (N = 3).

(D) Control and MCCC1 knockdown HeLa cells were either left untreated, AA starved for 2 hr, or AA starved and then Leu was added for 0.5 hr, then immunostained with mTOR and LAMP1 antibodies as shown. Co-localization panels show an overlap between mTOR and LAMP1 signals. The fraction of mTOR-positive lysosomes were determined using Volocity software. Values are mean ± SEM. n = ~50 cells. *$p < 0.05$, **$p < 0.01$ versus control cells; ##$p < 0.01$ versus AA-starved cells (two-tailed t test); ns, not significant. Scale bars, 5 μm and 1 μm (enlarged images). The experiment was repeated an additional two times (N = 3). NC, normal control.

(E) Immunoblots of control and MCCC1-knockdown HeLa cells with or without Leu or alpha-ketoisocaproate (KIC) under AA-starved conditions. Blots are representative of at least three independent experiments (N = 3). *$p < 0.05$, **$p < 0.01$, ***$p < 0.001$ versus control cells; #$p < 0.05$, ##$p < 0.01$ versus AA-starved cells; &&$p < 0.01$ versus MCCC1 knockdown cells. p-ULK1 (Ser757).

(F) Immunoblots of control and MCCC1-knockdown HeLa cells with or without 3-hydroxy-3-methylglutaryl-coenzyme A (HCoA) or AcCoA. *$p < 0.05$, **$p < 0.01$ versus control cells; #$p < 0.05$, ##$p < 0.01$ versus AA-starved cells. N = 3.

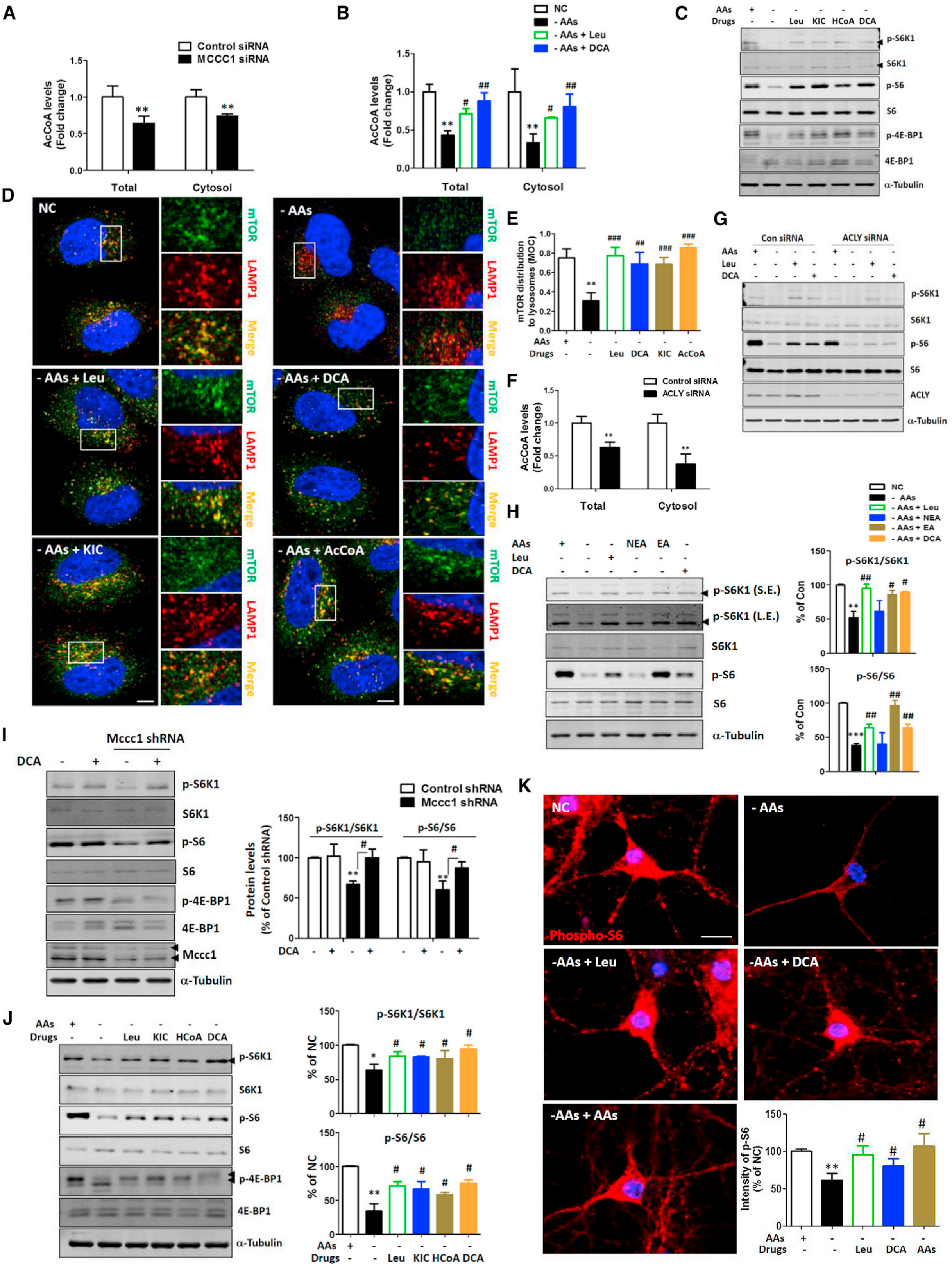

(legend on next page)

in a Rag complex-dependent manner without altering the localization of Rag complex. To determine whether known Leu sensors are required for mTORC1 activity rescue by AcCoA in AA-starved HeLa cells, we knocked down Sestrin (Sestrin 1, 2), LARS, and GLUD1, and found that mTORC1 activity was restored by DCA and partially by Leu (Figures S3C and S3D) in all cases. While we cannot exclude that complete knockout of these sensors would not have an effect, our data suggested that partial loss of these sensors is not limiting for the pathway we have described in HeLa cells, although the slightly blunted response to Leu in the Leu sensor knockdown cells may reflect roles for these pathways in addition to AcCoA biogenesis from Leu in regulating mTORC1 activity after starvation.

mTORC1 Regulation by AcCoA Is Associated with EP300 Acetyltransferase

To determine the exact mechanism by which AcCoA regulates mTORC1 signal pathway under AA starvation conditions, we checked acetylation levels in HeLa cells because AcCoA can activate lysine (K) acetyltransferases (KATs) (Marino et al., 2014; Pietrocola et al., 2015a). We found that starvation decreased acetylation levels globally, and the treatment with Leu and AcCoA rescued it (Figure S3E). We found that the acetylation in the cytoplasm particularly was reduced significantly, and MCCC1 knockdown mimicked starvation conditions (Figure S3F). Next, we investigated how alteration in acetylation levels by AcCoA could regulate mTORC1. Because EP300 acetyltransferase has been reported to regulate mTORC1 signaling by AcCoA (Marino et al., 2014), we treated cells with the specific EP300 inhibitors c646 and spermidine (Pietrocola et al., 2015b). We found that these compounds or EP300 knockdown blocked the ability of AcCoA to rescue mTORC1 activity in AA-starved HeLa cells (Figures 3A and 3B). However, knockdown of other common acetyltransferases, KAT2A and KAT2B, did not block the effect of AcCoA on mTORC1 activity under AA starvation (Figure 3C). Consistent with these data, the EP300 activator CTB, a simplified analog of CTPB, rescued mTORC1 inhibition by AA starvation (Figure S3G). To determine whether the acetyltransferase activity of EP300 is important for mTORC1 regulation, we used an EP300 acetyltransferase activity-dead mutant (EP300 Mut), and found that EP300 Mut failed to rescue mTORC1 signal by AcCoA in EP300 knockdown cells, while rescue was seen with the wild-type (EP300 WT) protein (Figure 3B). Likewise, immunostaining showed that EP300 knockdown prevented the lysosomal relocalization of mTORC1 in response to AcCoA (Figure 3D). EP300 did not appear to regulate mTORC2 activity (assessed by phosphorylation of PKCα/β or AKT) (Figure 3E).

To investigate how starvation regulates EP300 activity, we measured EP300 activity directly in total lysates and found that starvation for 2 hr did not alter total EP300 activity (Figure S3H). Consistent with previous reports showing that starvation altered EP300 localization (Sebti et al., 2014), we found that EP300 was located both in the nucleus and cytoplasm in basal conditions, and that AA starvation caused it to relocalize predominantly into the nucleus (Figure 3F). Likewise, AA starvation decreased the activity of cytoplasmic EP300 (Figure 3G). The addition of Leu and AcCoA to AA-starved cells caused a partial redistribution of EP300 into the cytoplasm from the nucleus (Figure 3H). Consistent with these data, AA starvation increased the acetylation of histone H3, a nuclear EP300 substrate, while acetylation of a cytoplasmic EP300 substrate, ATG7, was decreased (Figure 3I). These data indicate that mTORC1 regulation by AcCoA under AA starvation conditions is associated with changes in cytoplasmic EP300 acetyltransferase activity.

Raptor Acetylation by EP300 Is Important for mTORC1 Regulation

Having established that EP300 is able to modulate mTORC1 activity and its translocation to the lysosomes (Figure 3), we next investigated the mechanism for this effect. EP300 small interfering RNA or chemical inhibition did not alter the localization of Rag complex on the lysosome (Figure S3I), suggesting that EP300 acted upstream of this complex and that it may interact directly with mTORC1. We therefore assessed which component of mTORC1 bound EP300, and found that only Raptor interacted with it (Figures 4A, S4A, and S4B) independently of EP300 activity (Figure S4B). Furthermore, we were able to demonstrate that

Figure 2. AcCoA, the Metabolite of Leu Metabolism, Is Important for mTORC1 Regulation in Some Cell Types

(A) Measurement of total and cytosolic AcCoA levels in control and MCCC1 knockdown HeLa cells. $^{**}p < 0.01$ versus control cells. N = 3.

(B) Measurement of total and cytosolic AcCoA levels on HeLa cells with or without Leu or DCA under AA-starved conditions. $^{**}p < 0.01$ versus control cells; $^{\#}p < 0.05$, $^{\#\#}p < 0.01$ versus AA-starved cells. N = 4.

(C) mTORC1 regulation under AA-starved conditions with or without Leu, KIC, HCoA, or DCA in HeLa cells. Blots are representative of at least three independent experiments (N = 3).

(D) mTORC1 distribution to lysosomal membranes under AA starvation with or without Leu, KIC, DCA, or AcCoA in HeLa cells. Scale bars, 5 μm and 1 μm (enlarged images).

(E) Quantification of (D). Values are mean ± SEM. n = about 60 cells. $^{**}p < 0.01$ versus control cells; $^{\#\#}p < 0.01$, $^{\#\#\#}p < 0.001$ versus AA-starved cells (two-tailed t test).

(F) Measurement of total and cytosolic AcCoA levels in control and ACLY knockdown HeLa cells. $^{**}p < 0.01$ versus control cells. N = 3.

(G) Immunoblots of control and ACLY knockdown HeLa cells with or without Leu or DCA under AA-starved conditions. Blots are representative of at least three independent experiments. N = 3.

(H) mTORC1 regulation under AA-starved conditions with or without Leu or DCA in SH-SY5Y cells. $^{**}p < 0.01$, $^{***}p < 0.001$ versus control cells; $^{\#}p < 0.05$, $^{\#\#}p < 0.01$ versus AA-starved cells (two-tailed t test). NC, normal control; NEA, non-essential AA mixture; EA, essential AA mixture. N = 4.

(I) The role of DCA in Mccc1 knockdown primary neurons. $^{**}p < 0.01$ versus control small hairpin RNA (shRNA)-infected neurons; $^{\#}p < 0.05$ versus Mccc1 shRNA-infected neurons. N = 3.

(J) mTORC1 regulation under starved condition with or without Leu, KIC, HCoA, or DCA on primary neurons. $^{*}p < 0.01$, $^{**}p < 0.01$ versus control neurons; $^{\#}p < 0.05$ versus starved neurons. N = 3.

(K) mTORC1 regulation using phosphorylated S6 (p-S6; Ser235/236) antibody in primary neurons. Values are mean ± SEM. n = about 50 cells. $^{**}p < 0.01$ versus control neurons; $^{\#}p < 0.05$ versus starved neurons (two-tailed t test). Scale bar, 10 μm.

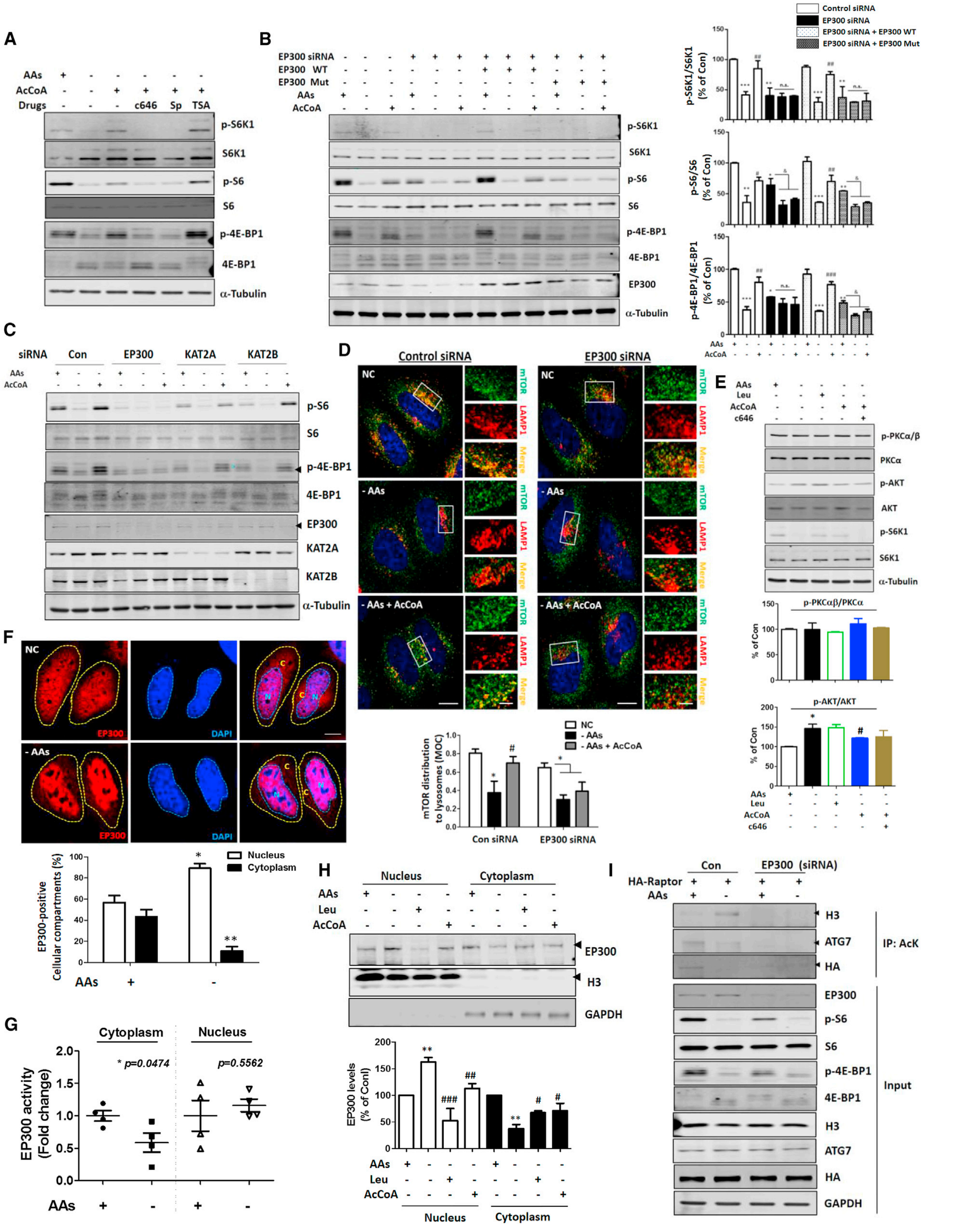

(legend on next page)

Raptor is acetylated, and that this acetylation was reduced by AA depletion (Figures 3I and 4B) or EP300 inhibition with c646 (Figures S4C and S4D), while the EP300 activator CTB increased Raptor acetylation (Figure S4E). Consistent with Figure 3C, neither KAT2A nor KAT2B induced Raptor acetylation (Figure 4C). We next determined which site in Raptor could be acetylated to regulate mTORC1. A previous study showed that K840 in Raptor can be acetylated and can regulate mTORC1 under specific conditions (Ma et al., 2015). However, the Raptor K840R mutant did not alter mTORC1 activity in AA-starved cells with/without AcCoA (Figure S4F) and, unexpectedly, mutation of K840 in Raptor did not prevent its acetylation in HeLa cells (Figure S4G). Recent liquid chromatography-mass spectrometry data showed that K1097 in Raptor can be acetylated (Lundby et al., 2012). We found that Raptor K1097R (KR) mutant had reduced acetylation (Figure 4D) and cells where Raptor was knocked down and replaced with Raptor KR failed to restore the mTORC1 activity in AA starvation with AcCoA (Figure 4E). Raptor KR did not alter the formation of mTORC1 (Figure S4H), but bound less to the Rag complex (Figures 4F and 4G). Likewise, immunostaining showed that cells with Raptor KR had decreased mTORC1 on lysosomal membranes (Figure 4H).

AcCoA Levels and Acetylation of Raptor Are Decreased in Tissues of Fasted Mice

Finally, to confirm the relevance of this mTOR signaling pathway *in vivo*, we assessed it in the brains of fasted mice. As seen for AA starvation in primary neurons (Figures 2J, 2K, and S2A), brains of fasted mice showed reduced mTORC1 activity (Figure S4I). We were able to confirm that the levels of AcCoA, the levels of acetylated Raptor and ATG7, and EP300 activity were also decreased in mouse brains following fasting (Figures 4I, 4J, and S4J–S4M). In addition to brain tissue, fasted mouse liver and muscle both showed significantly decreased levels of acetylated Raptor and significantly reduced AcCoA in the muscle with a non-significant trend in liver (Figures 4I and 4J).

In conclusion, while previous studies have suggested that cells require Leu sensing mechanisms to transduce nutrient status to the machinery controlling mTORC1 activity (Zheng et al., 2016), our study finds that Leu can signal directly via its metabolite AcCoA in some cell types. Increased AcCoA levels, which are found when Leu is added to AA-starved cells, activate the acetylation of Raptor at K1097. This increases its interaction with the Rag complex compared with unacetylated Raptor KR, and thus enables mTORC1 activation (Figure S4N). Interestingly, this mechanism appears to be relevant in a variety of cell types, including neurons. However, it is not obvious in MEF cells and HEK293T cells, which have been used prominently in this field for identifying alternate Leu signaling pathways (Han et al., 2012; Wolfson et al., 2016). This raises the possibility of cell-type-specific modes of regulation of mTORC1.

Limitations of Study

While we observed that starvation of mice decreased levels of AcCoA and Raptor acetylation in some tissues including the brain, further work is required to determine which tissues respond in this way to nutrient deprivation *in vivo*, and if these responses are different in cancers. It will be important to assess the importance of this Leu-AcCoA-mTORC1 signaling pathway in different tissues *in vivo*, in relation to other Leu-sensing mechanisms. It would be interesting to test the speculation that these different pathways whereby Leu regulates mTORC1 may act with different kinetics in different tissues after starvation is initiated *in vivo*.

STAR★METHODS

Detailed methods are provided in the online version of this paper and include the following:

- KEY RESOURCES TABLE
- CONTACT FOR REAGENT AND RESOURCE SHARING
- EXPERIMENTAL MODEL AND SUBJECT DETAILS
  - Cell Lines
  - Primary Mouse Cortical Neurons and Primary Embryonic Fibroblasts (MEFs)
  - Animals
- METHOD DETAILS
  - Antibodies and Reagents
  - Transfection
  - Western Blot Analysis
  - Starvation for Primary Cortical Neurons

Figure 3. mTORC1 Is Regulated by AcCoA via EP300 Acetyltransferase

(A) EP300 inhibitors (c646 and spermidine [Sp]) inhibit AcCoA-induced rescue of mTORC1 activity in AA-starved HeLa cells. N = 4.

(B) Effects of EP300 knockdown with replacement with wild-type (EP300 WT) or catalytically inactive EP300 (EP300 Mut) on mTORC1 signaling in response to AA deprivation with or without AcCoA. *p < 0.05, **p < 0.01, ***p < 0.001 versus control cells; #p < 0.05, ##p < 0.01, ###p < 0.001 versus AA-starved cells; &p < 0.05 versus EP300 knockdown cells (two-tailed t test); ns, not significant. N = 3.

(C) mTORC1 regulation by AcCoA in control HeLa cells or in EP300, KAT2A, or KAT2B knockdown HeLa cells. Blots are representative of at least three independent experiments (N = 3).

(D) mTORC1 distribution onto lysosomal membranes on control and EP300 knockdown HeLa cells in response to AA deprivation with or without AcCoA. Values are mean ± SEM. n = about 50 cells. *p < 0.05 versus control cells; #p < 0.05 versus AA-starved cells (two-tailed t test). Scale bars, 5 μm and 1 μm (enlarged images).

(E) mTORC2 regulation by EP300 activity. *p < 0.05 versus control cells; #p < 0.05 versus AA-starved cells (two-tailed t test). N = 3. p-PKCα/β (Thr638/641), p-AKT (Ser473).

(F) Localization of EP300 to the nucleus under AA-starved conditions. N, nuclear; C, cytoplasm. n = about 30 cells. *p < 0.05, **p < 0.01 versus control cells. Scale bar, 5 μm.

(G) Nuclear or cytoplasmic EP300 activities under AA-starved condition. *p < 0.05 versus control cells. N = 4.

(H) Change in EP300 localization under AA starvation with or without Leu or AcCoA. **p < 0.01 versus control cells; #p < 0.05, ##p < 0.01, ###p < 0.001 versus AA-starved cells (two-tailed t test). N = 3.

(I) Differential regulation of acetylation of nuclear (H3) and cytoplasmic (ATG7 and Raptor) substrates by EP300 under basal and AA starvation conditions. N = 3.

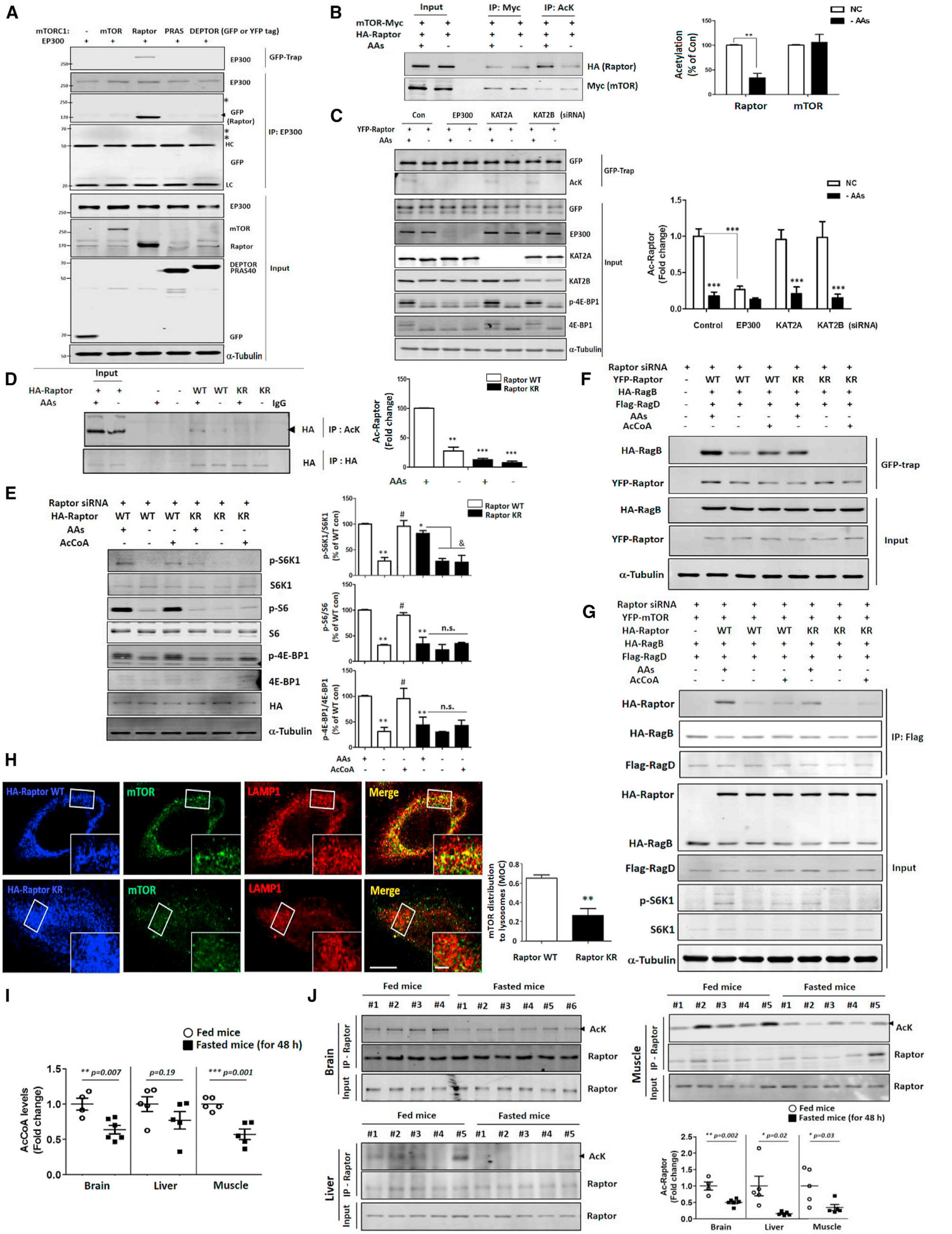

(legend on next page)

- shRNA Lentivirus Production and Infection
- Food Deprivation in Mice
- Mutagenesis
- Immunofluorescence
- Cytosolic/Nuclear Fractionation
- Acetyl-Coenzyme A (AcCoA) Measurement
- EP300 Activity Assay
- Co-Immunoprecipitation (Co-IP)
- GFP-Trap for IP of GFP (or YFP)-Fusion Proteins
- Mitochondrial Morphology Analysis
- Assessment of Oxidative Stress
- QUANTIFICATION AND STATISTICAL ANALYSIS
  - Image Analysis
  - Statistical Analysis
- DATA AND SOFTWARE AVAILABILITY

SUPPLEMENTAL INFORMATION

Supplemental Information includes four figures and one table and can be found with this article online at https://doi.org/10.1016/j.cmet.2018.08.013.

ACKNOWLEDGMENTS

We are grateful for funding from the UK Dementia Research Institute (funded by the MRC, Alzheimer's Research UK, and the Alzheimer's Society), Wellcome Trust (Principal Research Fellowship to D.C.R. [095317/Z/11/Z]), Strategic Grant to Cambridge Institute for Medical Research (100140/Z/12/Z), Roger de Spoelberch Foundation, Alzheimer's Research UK, Addenbrooke's Charitable Trust, National Institute for Health Research Cambridge Biomedical Research Centre (to D.C.R.), and the Korean Health Technology R&D Project, Ministry of Health & Welfare, Republic of Korea (HI14C2173 to S.M.S. and J.E.L.; HI14C1913 to H.L. and S.J.P.). The views expressed are those of the author(s) and not necessarily those of the NHS, the NIHR, or the Department of Health and Social Care.

AUTHOR CONTRIBUTIONS

S.M.S., F.M.M., J.E.L., and D.C.R. developed the study rationale. S.M.S. and D.C.R. wrote the manuscript, which was commented on by all authors. S.M.S. designed and performed most of the experiments. H.L. and F.M.M. generated constructs. S.J.P. and F.S. performed the experiments and assisted with data analysis. D.C.R. supervised the study.

DECLARATION OF INTERESTS

F.M.M. is currently employed by Eli Lilly.

Received: January 9, 2018
Revised: June 1, 2018
Accepted: August 12, 2018
Published: September 6, 2018

REFERENCES

Ashkenazi, A., Bento, C.F., Ricketts, T., Vicinanza, M., Siddiqi, F., Pavel, M., Squitieri, F., Hardenberg, M.C., Imarisio, S., Menzies, F.M., et al. (2017). Polyglutamine tracts regulate beclin 1-dependent autophagy. Nature *545*, 108–111.

Chantranupong, L., Wolfson, R.L., Orozco, J.M., Saxton, R.A., Scaria, S.M., Bar-Peled, L., Spooner, E., Isasa, M., Gygi, S.P., and Sabatini, D.M. (2014). The Sestrins interact with GATOR2 to negatively regulate the amino-acid-sensing pathway upstream of mTORC1. Cell Rep. *9*, 1–8.

Chu, C.H., and Cheng, D. (2007). Expression, purification, characterization of human 3-methylcrotonyl-CoA carboxylase (MCCC). Protein Expr. Purif. *53*, 421–427.

Dibble, C.C., and Manning, B.D. (2013). Signal integration by mTORC1 coordinates nutrient input with biosynthetic output. Nat. Cell Biol. *15*, 555–564.

Efeyan, A., Zoncu, R., and Sabatini, D.M. (2012). Amino acids and mTORC1: from lysosomes to disease. Trends Mol. Med. *18*, 524–533.

Han, J.M., Jeong, S.J., Park, M.C., Kim, G., Kwon, N.H., Kim, H.K., Ha, S.H., Ryu, S.H., and Kim, S. (2012). Leucyl-tRNA synthetase is an intracellular leucine sensor for the mTORC1-signaling pathway. Cell *149*, 410–424.

Hara, K., Yonezawa, K., Weng, Q.P., Kozlowski, M.T., Belham, C., and Avruch, J. (1998). Amino acid sufficiency and mTOR regulate p70 S6 kinase and eIF-4E BP1 through a common effector mechanism. J. Biol. Chem. *273*, 14484–14494.

He, X.D., Gong, W., Zhang, J.N., Nie, J., Yao, C.F., Guo, F.S., Lin, Y., Wu, X.H., Li, F., Li, J., et al. (2018). Sensing and transmitting intracellular amino acid signals through reversible lysine aminoacylations. Cell Metab. *27*, 151–166.e6.

Jewell, J.L., Russell, R.C., and Guan, K.L. (2013). Amino acid signalling upstream of mTOR. Nat. Rev. Mol. Cell Biol. *14*, 133–139.

Jimenez-Sanchez, M., Lam, W., Hannus, M., Sonnichsen, B., Imarisio, S., Fleming, A., Tarditi, A., Menzies, F., Dami, T.E., Xu, C., et al. (2015). siRNA screen identifies QPCT as a druggable target for Huntington's disease. Nat. Chem. Biol. *11*, 347–354.

Laplante, M., and Sabatini, D.M. (2012). mTOR signaling in growth control and disease. Cell *149*, 274–293.

Lorin, S., Tol, M.J., Bauvy, C., Strijland, A., Pous, C., Verhoeven, A.J., Codogno, P., and Meijer, A.J. (2013). Glutamate dehydrogenase contributes to leucine sensing in the regulation of autophagy. Autophagy *9*, 850–860.

Lundby, A., Lage, K., Weinert, B.T., Bekker-Jensen, D.B., Secher, A., Skovgaard, T., Kelstrup, C.D., Dmytriyev, A., Choudhary, C., Lundby, C.,

Figure 4. Raptor Acetylation by EP300 Is Important for mTORC1 Regulation

(A) Interaction of EP300 with components of the mTORC1 using GFP-trap or immunoprecipitation (IP) with EP300 antibody. GFP- or YFP-tagged mTORC1 constructs (GFP vector, YFP-mTOR, YFP-Raptor, YFP-proline-rich Akt substrate of 40 kDa [PRAS40], and YFP-DEP domain-containing mTOR-interacting protein [DEPTOR]) were used. Asterisks indicate the predicted position of mTOR, PRAS40, or DEPTOR. HC, heavy immunoglobulin G (IgG) chain; LC, light IgG chain. N = 3.

(B) Decreased acetylation of Raptor under AA-starved conditions independently of interaction with mTOR. Acetylated mTOR level was not altered by AA starvation. $^{**}p < 0.01$ versus control cells. N = 3.

(C) Acetylation of Raptor by EP300, not by KAT2A or KAT2B. $^{***}p < 0.001$ versus control cells. N = 3.

(D) Raptor K1097R mutant (KR) is not acetylated. WT, wild-type; KR, K1097R mutant. $^{**}p < 0.01$, $^{***}p < 0.001$ versus Raptor WT-expressing control cells. N = 3.

(E) Cells were depleted of Raptor with small interfering RNA (siRNA) and reconstituted with Raptor WT or KR, then analyzed for mTORC1 activity in the presence or absence of AA, the latter with/without AcCoA in HeLa cells. $^{*}p < 0.05$, $^{**}p < 0.01$ versus Raptor WT-expressing control cells; $^{\#}p < 0.05$ versus AA-starved cells; $^{\&}p < 0.05$ versus Raptor KR-expressing cells (two-tailed t test); ns, not significant. N = 3.

(F and G) Reduced interaction of Raptor KR mutant with the Rag complex using GFP-trap (F) or IP with Flag antibody (G). N = 3.

(H) mTORC1 distribution onto lysosomal membranes in HeLa cells depleted of Raptor then reconstituted with Raptor WT or Raptor KR. Scale bars, 10 μm and 2 μm (enlarged images). n = about 40 cells. $^{**}p < 0.01$ versus Raptor WT-expressing control cells.

(I and J) Decreased AcCoA levels and acetylation of Raptor in fasted mice brains, livers, and muscles. After 22.5 hr starvation, mice were given free access to food for 1.5 hr followed by a second round of starvation for another 22.5 hr. The tissue samples from fed (n = 5) and fasted mice (n = 6) were analyzed for AcCoA (I) and acetylated Raptor (J) levels. $^{*}p < 0.05$, $^{**}p < 0.01$, $^{***}p < 0.001$ versus fed mice.

et al. (2012). Proteomic analysis of lysine acetylation sites in rat tissues reveals organ specificity and subcellular patterns. Cell Rep. *2*, 419–431.

Ma, L., Tang, H., Yin, Y., Yu, R., Zhao, J., Li, Y., Mulholland, M.W., and Zhang, W. (2015). HDAC5-mTORC1 interaction in differential regulation of ghrelin and nucleobindin 2 (NUCB2)/nesfatin-1. Mol. Endocrinol. *29*, 1571–1580.

Marino, G., Pietrocola, F., Eisenberg, T., Kong, Y., Malik, S.A., Andryushkova, A., Schroeder, S., Pendl, T., Harger, A., Niso-Santano, M., et al. (2014). Regulation of autophagy by cytosolic acetyl-coenzyme A. Mol. Cell *53*, 710–725.

Parmigiani, A., Nourbakhsh, A., Ding, B., Wang, W., Kim, Y.C., Akopiants, K., Guan, K.L., Karin, M., and Budanov, A.V. (2014). Sestrins inhibit mTORC1 kinase activation through the GATOR complex. Cell Rep. *9*, 1281–1291.

Pietrocola, F., Galluzzi, L., Bravo-San Pedro, J.M., Madeo, F., and Kroemer, G. (2015a). Acetyl coenzyme a: a central metabolite and second messenger. Cell Metab. *21*, 805–821.

Pietrocola, F., Lachkar, S., Enot, D.P., Niso-Santano, M., Bravo-San Pedro, J.M., Sica, V., Izzo, V., Maiuri, M.C., Madeo, F., Marino, G., et al. (2015b). Spermidine induces autophagy by inhibiting the acetyltransferase EP300. Cell Death Differ. *22*, 509–516.

Sancak, Y., Bar-Peled, L., Zoncu, R., Markhard, A.L., Nada, S., and Sabatini, D.M. (2010). Ragulator-Rag complex targets mTORC1 to the lysosomal surface and is necessary for its activation by amino acids. Cell *141*, 290–303.

Sancak, Y., Peterson, T.R., Shaul, Y.D., Lindquist, R.A., Thoreen, C.C., Bar-Peled, L., and Sabatini, D.M. (2008). The Rag GTPases bind raptor and mediate amino acid signaling to mTORC1. Science *320*, 1496–1501.

Saxton, R.A., Knockenhauer, K.E., Wolfson, R.L., Chantranupong, L., Pacold, M.E., Wang, T., Schwartz, T.U., and Sabatini, D.M. (2016). Structural basis for leucine sensing by the Sestrin2-mTORC1 pathway. Science *351*, 53–58.

Saxton, R.A., and Sabatini, D.M. (2017). mTOR signaling in growth, metabolism, and disease. Cell *169*, 361–371.

Sebti, S., Prebois, C., Perez-Gracia, E., Bauvy, C., Desmots, F., Pirot, N., Gongora, C., Bach, A.S., Hubberstey, A.V., Palissot, V., et al. (2014). BAG6/BAT3 modulates autophagy by affecting EP300/p300 intracellular localization. Autophagy *10*, 1341–1342.

Wolfson, R.L., Chantranupong, L., Saxton, R.A., Shen, K., Scaria, S.M., Cantor, J.R., and Sabatini, D.M. (2016). Sestrin2 is a leucine sensor for the mTORC1 pathway. Science *351*, 43–48.

Zheng, L., Zhang, W., Zhou, Y., Li, F., Wei, H., and Peng, J. (2016). Recent advances in understanding amino acid sensing mechanisms that regulate mTORC1. Int. J. Mol. Sci. *17*, https://doi.org/10.3390/ijms17101636.

Zoncu, R., Efeyan, A., and Sabatini, D.M. (2011). mTOR: from growth signal integration to cancer, diabetes and ageing. Nat. Rev. Mol. Cell Biol. *12*, 21–35.

STAR★METHODS

KEY RESOURCES TABLE

REAGENT or RESOURCE	SOURCE	IDENTIFIER
Antibodies		
Rabbit polyclonal anti-Actin	Sigma Aldrich	#A2066; RRID: AB_476693
Rabbit polyclonal anti-AKT	Cell Signaling Technology	#9272; RRID: AB_329827
Rabbit monoclonal anti-phospho-AKT (p-AKT) (Ser473)	Cell Signaling Technology	#4060; RRID: AB_2315049
Rabbit monoclonal anti-ATG7 (D12B11)	Cell Signaling Technology	#8558; RRID: AB_10831194
Rabbit polyclonal anti-ATP-Citrate Lyase (ACLY)	Cell Signaling Technology	#4332; RRID: AB_2223744
Rabbit polyclonal anti-phospho-ATP-Citrate Lyase (p-ACLY)(Ser455)	Cell Signaling Technology	#4331; RRID: AB_2257987
Rabbit monoclonal anti-AUH	Abcam	#ab157453
Rabbit monoclonal anti-acetylated-Lysine (Ac-K)	Cell Signaling Technology	#9814; RRID: AB_10544700
Rabbit polyclonal anti-acetylated-Lysine (Ac-K)	Cell Signaling Technology	#9441; RRID: AB_331805
Rabbit polyclonal anti-4E-BP1	Cell Signaling Technology	#9452; RRID: AB_331692
Rabbit polyclonal anti-phospho-4E-BP1 (Thr37/46)	Cell Signaling Technology	#9459; RRID: AB_2262165
Rabbit polyclonal anti-EP300	SantaCruz Biotechnology	#sc-585; RRID: AB_2616339
Mouse monoclonal anti-EP300	Millipore	#05-257; RRID: AB_309670
Mouse monoclonal anti-Flag M2	Sigma Aldrich	#F3165; RRID: AB_259529
Mouse monoclonal anti-GAPDH clone 6C5	Abcam	#ab8245; RRID: AB_2107448
Mouse monoclonal anti-GFP	Clontech	#632375
Mouse monoclonal anti-GFP	Clontech	#632592; RRID: AB_2336883
Mouse monoclonal anti-HA.11 clone 16B12	Covance	#MMS-101P; RRID: AB_2314672
Goat polyclonal anti-HA	Novus Biologicals	#NB600-362; RRID: AB_10124937
Rabbit polyclonal anti-Histone H3	Cell Signaling Technology	#9715; RRID: AB_331563
Mouse monoclonal anti-LAMP1 clone H4A3	Developmental Studies Hybridoma Bank, University of Iowa	N/A
Rabbit monoclonal anti-LAMP1	Cell Signaling Technology	#9091; RRID: AB_2687579
Rabbit monoclonal anti-mLST8 (86B8)	Cell Signaling Technology	#3274; RRID: AB_823685
Rabbit monoclonal anti-LAMTOR1/C11orf59	Cell Signaling Technology	#8975; RRID: AB_10860252
Rabbit polyclonal anti-MCCC1	Novus Biologicals	#NBP1-81254; RRID: AB_11033675
Mouse monoclonal anti-c-Myc clone 9E10	Roche	#11667203001; RRID: AB_390911
Rabbit polyclonal anti-total p70S6kinase (S6K1)	Cell Signaling Technology	#9202; RRID: AB_331676
Rabbit monoclonal anti-phospho-p70S6kinase (p-S6K1) (Thr389)	Cell Signaling Technology	#9234; RRID: AB_2269803
Rabbit monoclonal anti-PRAS40 (D23C7)	Cell Signaling Technology	#2691; RRID: AB_2225033
Rabbit polyclonal anti-PKCα	Cell Signaling Technology	#2056; RRID: AB_2284227
Rabbit polyclonal anti-phospho-PKCα/β II (Thr638/641)	Cell Signaling Technology	#9375; RRID: AB_2284224
Goat polyclonal anti-Raptor	SantaCruz Biotechnology	#sc-27744; RRID: AB_2130790
Rabbit polyclonal anti-phospho-Raptor (Ser863)	Signalway	#12778
Rabbit monoclonal anti-Raptor (24C12)	Cell Signaling Technology	#2280; RRID: AB_561245
Rabbit polyclonal anti-phospho-Raptor (Ser792)	Cell Signaling Technology	#2083; RRID: AB_2249475
Rabbit monoclonal anti-RagA (D8B5)	Cell Signaling Technology	#4357; RRID: AB_10545136
Rabbit monoclonal anti-RagB	Cell Signaling Technology	#8150; RRID: AB_11178806
Rabbit monoclonal anti-RagC (D8H5)	Cell Signaling Technology	#9480; RRID: AB_10614716
Rabbit monoclonal anti-S6 Ribosomal Protein (S6)	Cell Signaling Technology	#2217; RRID: AB_331355
Rabbit anti-phospho-S6 Ribosomal Protein (p-S6) (Ser235/236)	Cell Signaling Technology	#4856; RRID: AB_2181037
Mouse monoclonal anti-α-Tubulin	Sigma Aldrich	#T9026; RRID: AB_477593
Rabbit polyclonal anti-mTOR	Cell Signaling Technology	#2972; RRID: AB_330978

(*Continued on next page*)

Continued

REAGENT or RESOURCE	SOURCE	IDENTIFIER
Rabbit polyclonal anti-phospho-mTOR (Ser2481)	Cell Signaling Technology	#2974; RRID: AB_2262884
Rabbit polyclonal anti-ULK1	Cell Signaling Technology	#4773; RRID: AB_2288252
Rabbit anti-phospho-ULK1 (Ser757)	Cell Signaling Technology	#6888; RRID: AB_10829226
Chemicals, Peptides, and Recombinant Proteins		
hEGF	Sigma Aldrich	#E9644
Hydrocortisone	Sigma Aldrich	#H0135
Cholera toxin	Sigma Aldrich	#C8052
Insulin	Sigma Aldrich	#I9278
KIC	Sigma Aldrich	#K0629
NAC	Sigma Aldrich	#A7250
DCA	Sigma Aldrich	#347795
HCoA	Sigma Aldrich	#H6132
AcCoA	Sigma Aldrich	#A2056
c646	Sigma Aldrich	#SML0002
Spermidine	Sigma Aldrich	#S0266
Potassium hydroxycitrate tribasic monohydrate (HC)	Sigma Aldrich	#59847
TSA	Sigma Aldrich	#T8552
MitoTracker green FM	Invitrogen	# M7514
MitoSOX Red reagent	Molecular Probes	#M36008
Critical Commercial Assays		
PicoProbe AcCoA assay kit	Abcam	#ab87546
SensoLyte EP300 assay kit	Anaspec	#AS-72172
GFP-TRAP	Chromotek	Gtma-20
Experimental Models: Cell Lines		
HeLa	ATCC	#CCL-2; CVCL_0030
HEK-293T	ATCC	#CRL-3216; CVCL_0063
MCF7	ATCC	#HTB-22; CVCL_0031
H4	ATCC	#HTB-148; CVCL_1239
MCF10A	Horizon	#HD PAR-058
MSC	ATCC	#PCS-500-011
Primary mouse cortical neurons	in-house	N/A
MEF	in-house	N/A
Experimental Models: Organisms/Strains		
C57BL/6 mice	Jackson Laboratories	C57BL/6
Oligonucleotides		
Primer for Raptor K1097R forward primer, 5'-ccaaatcagcaaa attcctccagaccctgatggca-3'	This paper	N/A
Primer for Raptor K1097R reverse primer, 5'-tgccatcagggtct ggaggaattttgctgatttgg-3'	This paper	N/A
Primer for Raptor K840R forward primer, 5'-cacggtggccctgt aggcgatgctgttgag-3'	This paper	N/A
Primer for Raptor K840R reverse primer, 5'-ctcaacagcatcgc ctacagggccaccgtg-3'	This paper	N/A
For siRNA sequences see Table S1	N/A	N/A
Recombinant DNA		
pEFGP	Clontech	
pCMV6-XL5-MCCC1	Origene	#SC113201
pcDNA3.1-EP300	Addgene	#23252
pcDNA3.1-EP300(HAT-)	Addgene	#23254

(*Continued on next page*)

REAGENT or RESOURCE	SOURCE	IDENTIFIER
Continued		
peYFP-C1-mTOR	Addgene	#73384
pRK5-HA-YFP-Raptor	Addgene	#73385
pRK5-HA-YFP-PRAS40	Addgene	#73389
pRK5-HA-YFP-DEPTOR	Addgene	#73390
pRK5-HA-Raptor	Addgene	#8513
pRK5-HA-GST-RagB	Addgene	#19301
pLJM1-Flag-RagD	Addgene	#19316
pLJM1-Flag-Raptor-Rheb15	Addgene	#26634
pLKO.1 shRNAs	Dharmacon	#RHS4080
mouse Mccc1 shRNA	Dharmacon	#RMM4534-EG72039
Software and Algorithms		
Prism	GraphPad	https://www.graphpad.com/scientific-software/prism/
ImageJ	Fiji	http://fiji.sc/
IMAGE STUDIO Lite	LI-COR Biosciences	https://www.licor.com/bio/products/software/image_studio_lite/
Volocity	PerkinElmer	http://cellularimaging.perkinelmer.com/
Photoshop	Adobe	http://www.adobe.com/uk/products/photoshop.html
Other		
DMEM	Sigma Aldrich	#D6546
FBS	Sigma Aldrich	#F7524
FBS, dialyzed	Thermo Fisher Scientific	#26400-044
L-glutamine	Sigma Aldrich	#G7513
Penicillin/ streptomycin	Sigma Aldrich	#P0781
Horse serum	Sigma Aldrich	#H1270
Hank’s balanced salt solution	Sigma Aldrich	#H9394
Earle’s balanced salt solution	Sigma Aldrich	#E7510
leucine	Sigma Aldrich	#L8000
isoleucine	Sigma Aldrich	#I2752
valine	Sigma Aldrich	#V0500
Neurobasal-A medium	Thermo Fisher Scientific	#12349015

CONTACT FOR REAGENT AND RESOURCE SHARING

Further information and requests for resources and reagents should be directed to and will be fulfilled by the Lead Contact, David C. Rubinsztein (dcr1000@cam.ac.uk).

EXPERIMENTAL MODEL AND SUBJECT DETAILS

Cell Lines

Human cervical epithelium HeLa, human neuroblastoma cell line SH-SY5Y, human embryonic kidney cell line HEK-293T, human breast cancer cell line MCF7 and human neuroglioma cell line H4 cells (from ATCC) cultured in Dulbecco’s modified Eagle’s medium (DMEM) (4.5 mg l^{-1} of glucose; #D6546)) supplemented with 10% FBS (#F7524), 2mM L-glutamine (#G7513) and 100Uml^{-1} penicillin and 100 mgml^{-1} streptomycin (#P0781). Non-transformed mammary epithelial MCF10A cells were purchased from Horizon (Catalogue No. HD PAR-058) and cultured in DMEM-F12 supplemented with 5% horse serum (#H1270), 20 ngml^{-1} hEGF (#E9644), 0.5 μgml^{-1} hydrocortisone (#H0135), 100 ngml^{-1} cholera toxin (#C8052), 10 μgml^{-1} insulin (#I9278) and 100 Uml^{-1} penicillin and 100 mgml^{-1} streptomycin (all the components were obtained from Sigma Aldrich). A human adipose-derived Mesenchymal Stem Cells (MSC) cell line was purchased from ATCC (#PCS-500-011), and maintained in Mesenchymal Stem Cell Basal Medium (ATCC, #PCS-500-030), supplemented using the Mesenchymal Stem Cell Growth Kit (ATCC, #PCS-500-040), according to the manufacturer’s recommendation. All the cell lines were maintained at 37°C and 5% CO_2 and were regularly tested for mycoplasma contamination. HeLa, SHSY5Y, HEK-293T, MCF7 and MCF10A cells are female, and H4 cells are male. For starvation experiments

with cell lines, cells were washed three times in Hank's balanced salt solution (Invitrogen) and incubated for 2 h at 37°C in Earle's balanced salt solution (Starvation media) (Sigma-Aldrich). All experiments with amino acid starvation and stimulation contained 10% dialyzed FBS (#26400-036 from Invitrogen) unless otherwise indicated. The cells were stimulated with essential amino acid (AAs) at a concentration 2X of what is typically found in normal Invitrogen high-glucose DMEM.

Primary Mouse Cortical Neurons and Primary Embryonic Fibroblasts (MEFs)

Primary cortical neurons were isolated from C57BL/6 mice (Jackson Laboratories) embryos at E16.5 as described previously (Jimenez-Sanchez et al., 2015). Briefly, brains were harvested and placed in HBSS where the meninges were removed and the cerebral cortices were dissected. After incubation in Hank's Balanced Salt Solution (HBSS) with 0.25% trypsin (Gibco) for 20 min at 37°C, dissociated neurons were resuspended in HBSS and seeded on poly-D-lysine-coated 6- or 12-multiwell plates. Cells were cultured in maintenance media (Neurobasal-A medium (#12349015, Thermo Fisher Scientific) supplemented with 2 mM GlutaMAX, 200 mM B27 supplement and 1% Penicillin–Streptomycin) at 37°C in a humidified incubator with 5% CO_2. One half of the culture medium was changed every 2 days until treatment/infection. After 5 days of *in vitro* culturing, differentiated neurons were infected with lentiviral particles for knockdown experiments. Primary mouse embryonic fibroblasts (MEFs) were isolated from wild-type C57BL/6 mice embryos at E12, using a protocol modified from that described previously (http://www.thermofisher.com/). The sexes of mouse-derived primary cells were not determined, as this was not considered to impact the basic cell biology we are describing. All mouse experiments were performed with personal and project licences granted by the UK Home Office and with the approval of the University of Cambridge committee for animal studies.

Animals

For all studies, 6 to 7-weeks-old C57BL/6 male or female mice were used, and all studies were performed under the jurisdiction of appropriate Home Office Project and Personal animal licences and with local Ethics Committee approval from the University of Cambridge. The mice used in this study for obtaining the brains, livers and muscles were group-housed and maintained in cages with standard bedding and enrichment. Mice were maintained in a temperature and humidity-controlled room on a 12 h light cycle with ad libitum access to water and standard laboratory chow diet. Water and cages were autoclaved. The ratio of sexes of used mice was 1:1 (for 48 h fasting) or only females were used for 23 h fasting experiment. In general, n=4-6 mice were used per group and experiments were repeated at least twice. The number of the mice used for the experiments are indicated for each experiment in the figure legends. No other inclusion or exclusion criteria were used. No significant differences of AcCoA levels or acetylated Raptor levels between sexes were observed.

METHOD DETAILS

Antibodies and Reagents

The following antibodies have been used in this work: mouse anti-Flag M2 (#F3165), rabbit anti-Actin (#A2066) and mouse anti-α-Tubulin (#T9026) from Sigma Aldrich; mouse anti-GAPDH clone 6C5 (#ab8245) and rabbit anti-AUH (#ab157453) from Abcam; rabbit anti-MCCC1 (#NBP1-81254; Novus Biologicals); goat anti-Raptor (#sc-27744) and rabbit anti-EP300 (#sc-585) from SantaCruz Biotechnology; mouse anti-GFP (#632375 and #632592; Clontech); rabbit anti-phospho-Raptor (Ser863; #12778; Signalway); mouse anti-HA.11 clone 16B12 (#MMS-101P, Covance); mouse anti-EP300 (#05-257) from Millipore; mouse anti-LAMP1 clone H4A3 (obtained from Developmental Studies Hybridoma Bank, University of Iowa); mouse anti-c-Myc clone 9E10 (#11667203001; Roche); rabbit anti-LAMP1 (#9091), rabbit anti-mTOR (#2972), rabbit anti-phospho-mTOR (Ser2481; #2974), rabbit anti-phospho-p70S6kinase (p-S6K1) (Thr389; #9234), anti-total p70S6kinase (S6K1) (#9202), rabbit anti-phospho-S6 Ribosomal Protein (p-S6) (Ser235/236; #4856), rabbit anti-S6 Ribosomal Protein (S6) (#2217), rabbit anti-phospho-4E-BP1 (Thr37/46; #9459), rabbit anti-4E-BP1 (#9452), rabbit anti-phospho-ULK1 (Ser757; #6888), rabbit anti-ULK1 (#4773), rabbit anti-phospho-Raptor (Ser792; #2083), rabbit anti-Raptor (24C12) (#2280), rabbit anti-acetylated-Lysine (Ac-K) (#9814, #9441), rabbit anti-Histone H3 (#9715), rabbit anti-ATG7 (D12B11) (#8558), rabbit anti-PRAS40 (D23C7) (#2691), rabbit anti-mLST8 (86B8) (#3274), rabbit anti-LAMTOR1/C11orf59 (#8975), rabbit anti-RagA (D8B5) (#4357), rabbit anti-RagB (#8150) and rabbit anti-RagC (D8H5) (#9480), anti-phospho-PKCα/β II (Thr638/641; #9375), anti-PKCα (#2056), anti-phospho-AKT (p-AKT) (Ser473;#4060), anti-AKT (#9272), anti-phospho-ATP-Citrate Lyase (p-ACLY)(Ser455; #4331), anti-ATP-Citrate Lyase (ACLY) (#4332) from Cell Signaling Technology; anti-mouse (#NA931V) and anti-rabbit (#NA934V) horseradish peroxidise (HRP)-conjugated secondary antibodies (GE Healthcare); anti-goat horseradish peroxidise (HRP)-conjugated secondary antibody (#611620, Invitrogen/Life Technologies). All the primary antibodies were used at a dilution of 1:1,000 (overnight incubation at 4°C), unless otherwise stated, and the secondary antibodies used at a dilution between 1:2,000 and 1:4,000 (1 h of incubation at room temperature).

Drug treatments used include: DMSO, .1-10 μM leucine, .1-100 μM isoleucine, .1-100 μM valine, 1-10 μM KIC, 1 mM NAC, .1-1 mM DCA, 1-10 μM HCoA, .1-1 μM AcCoA, 10 μM c646, 10 μM Spermidine, 50 μM CTB, 20mM Potassium hydroxycitrate tribasic monohydrate (HC), 50 μM TSA from Sigma-Aldrich.

Transfection

Trans IT-2020 reagent (Mirus) was used for DNA transfection, while Lipofectamine 2000 (Invitrogen) was used for siRNA transfections, according to the manufacturer's instructions. After transfection, cells were maintained in full medium. For knockdown

experiments, unless otherwise specified in figure legends, cells were transfected with 50 nM siRNA followed by another 50 nM siRNA transfection after 48 h. Cells were split once in between both transfections and harvested after 3 days of transfection. The following DNA or siRNA/shRNA constructs were also used in this work: empty pEFGP and pcDNA3.1-myc-His (Invitrogen); pCMV6-XL5-MCCC1 (untagged) (#SC113201) from Origene; pcDNA3.1-p300 (#23252), pcDNA3.1-p300(HAT-) (#23254), peYFP-C1-mTOR (#73384), pRK5-HA-YFP-Raptor (#73385), pRK5-HA-YFP-PRAS40 (#73389), pRK5-HA-YFP-DEPTOR (#73390), pRK5-HA-Raptor (#8513), pRK5-HA-GST-RagB (#19301), pLJM1-Flag-RagD (#19316), pLJM1-Flag-Raptor-RHEB15 (#26634) from Addgene. Pre-designed siRNAs (SMARTpool and/or set of deconvoluted oligos ON-TARGETplus4Non-targeting Control #D-001810-10; RagA #L-016070-00-0005, RagB #L-012189-01-0005, LAMTOR1 #L-020916-02-0005, AUH #L-008457-00-0005, HMGCL #L-019290-00-0005, KAT2A #L-009722-02-0005, KAT2B #L-005055-00-0005, KAT5 #L-006301-00-0005, KAT8 #L-014800-02-0005, EP300 #L-003486-00-0005, Raptor #L-004107-00-0005, Sestrin 1 #L-020244-00-0005, Sestrin 2 #L-019134-02-0005, LARS #L-010171-00-0005, ACLY #L-004915-00-0005) were obtained from Dharmacon-Thermo Scientific; For knockdown of MCCC1, we used siRNA from Ambion (#AM51331, s32399, s32400, s32401) as well as from Dharmacon-Thermo Scientific (#L-009429-00-0005). For knock-down of GLUD1, we used siRNA from Ambion (#AM16708, 107673). Pre-designed pLKO.1 shRNAs vectors from The RNAi Consortium (TRC) (empty vector control #RHS4080, Dharmacon; mouse Mccc1 #RMM4534-EG72039). Although all the shRNAs provided by TRC were validated, we used to perform most of the experiments (TRCN0000112506 (#B)).

Western Blot Analysis

The tissue samples were lysed in tissue lysis buffer on ice (20 mM Tris-HCl (pH7.4), 5 mM EDTA, 150 mM NaCl, 0.5% Triton X-100, 10 mM sodium butyrate, 1 mM TSA and protease/phosphatase inhibitors cocktail) and the supernatant was centrifuged twice. Protein concentration was determined using the Bradford assay (Bio-Rad). Cells were lysed in Laemmli buffer and protein samples were boiled for 10 min at 100°C, separated by SDS-PAGE, transferred onto PVDF membranes, subjected to western blot analysis, finally visualized using an ECL enhanced chemiluminescence detection kit (GE Healthcare), or with direct infrared fluorescence detection on an Odyssey Infrared Imaging System. Densitometric analysis on the immunoblots was performed using ImageJ program or IMAGE STUDIO Lite software, which enables quantitative analysis of blotting signals.

Starvation for Primary Cortical Neurons

For starvation experiments with primary neurons, neurons were washed twice in either maintenance media or artificial CSF (ACSF) (10 mM HEPES, pH 7.4, 125 mM NaCl, 5 mM KCl, 2 mM $CaCl_2$, 1 mM $MgCl_2$, 10 mM glucose) and incubated in maintenance media or ACSF for 4 h.

shRNA Lentivirus Production and Infection

shRNA lentiviral particles were produced and transduced following The RNAi Consortium (TRC) protocols. Briefly, HEK-293T packaging cells growing in 100 mm dishes were transfected at 60–70% of confluence with a mix of 2.5 μg psPAX2 vector (packaging vector), 270 ng pMD2.G vector (envelope vector) and 2.7 μg hairpin-pLKO.1 vector. TransIT-LT1 (Mirus) was used as transfection reagent according to the manufacturer's instructions. After transfection, cells were cultured in high-serum medium (20% FBS). Cell culture medium was harvested three times for intervals of 24 h. Viral preps were then concentrated by centrifugation at 160,100g for 90 min. For primary neurons, 20 μl of viral preps were added to the cells in the presence of 4 μg/ml polybrene (Sigma Aldrich) and were incubated for 24 h. The medium was replaced by full medium and cells were further incubated for an additional 4 days before testing the knockdown effects.

Food Deprivation in Mice

All animal studies were performed under the jurisdiction of appropriate Home Office Project and Personal animal licences and with local Ethics Committee approval from the University of Cambridge. For fasting, we used two different procedures. Either, the 6-weeks-old C57BL/6 male or female mice were deprived of food for 48 h with free access to water throughout the procedure, as described previously (Ashkenazi et al., 2017). After 22.5 h starvation, mice were given free access to food for 1.5 h followed by a second round of starvation for another 22.5 h. Our rationale is that this procedure synchronises the starvation. Alternatively, mice were deprived of food for 23 h with free access to water throughout the procedure. The tissues were collected and frozen for immunoblot and immunoprecipitation analysis.

Mutagenesis

Mutagenesis of the human Raptor was generated with QuikChange Site-Directed Mutagenesis Kit (Agilent Stratagene) according to manufacturer's instructions, using the set of primers:

1) 5'-ccaaatcagcaaaattcctccagaccctgatggca-3' (forward), 5'-tgccatcagggtctggaggaattttgctgatttgg-3' (reverse) to convert the residue Lys1097 into an Arg (K1097R)
2) 5'-cacggtggccctgtaggcgatgctgttgag-3' (forward), 5'-ctcaacagcatcgcctacagggccaccgtg-3' (reverse) to convert the residue Lys840 into an Arg (K840R)

DPN I digestion was performed after PCR and XL-10 gold-competent cells were transformed, and through sequencing, Raptor mutants from a positive clone were selected.

Immunofluorescence

For immunofluorescence, the HeLa, HEK-293T or primary neurons were fixed for 5 min with ice cold methanol or for 10 min with 4% paraformaldehyde (PFA). Concentration of the primary and secondary antibodies are described below. The mounting solution was from Molecular Probes.

Dilution of primary antibodies. 1:300 rabbit anti-mTOR; 1:600 mouse anti-LAMP1; 1:300 anti-phospho-S6 Ribosomal Protein; 1:400 rabbit anti-acetylated-Lysine (Ac-K); 1:500 rabbit anti-RagC; 1:200 rabbit anti-EP300; 1:200 goat anti-Raptor; 1:600 goat anti-HA (#NB600-362; Novus Biologicals); 1:1000 mouse anti-HA.11 clone 16B12.

The secondary antibodies Alexa 488, 555, 568, 594 or 647 goat anti-mouse, goat anti-rabbit or rabbit anti-goat were obtained from Molecular Probes and used at 1:400. Imaging was conducted with LSM780 Zeiss confocal with 63X oil-immersion lense. The colocalization was measured using Volocity software for Mander's Overlap Coefficient (MOC) or Pearson's correlation coefficient (PCC). These procedures were performed in a blinded fashion.

Cytosolic/Nuclear Fractionation

Cells were washed twice with ice-cold PBS and lysed with Buffer A (10 mM HEPES, 10 mM KCl, 0.1 mM EDTA, 0.4% NP-40, 1 mM DTT and protease/phosphatase inhibitors cocktail) and incubated on ice for 20 min. After homogenization, the lysates were centrifuged at 16,100g at 4°C for 10 min. Supernatants containing cytosolic proteins were harvested and nuclear pellets were resuspended with Buffer B (20 mM HEPES, 0.4 M NaCl, 1 mM EDTA, 10 % glycerol, 1 mM DTT and protease/phosphatase inhibitors cocktail) and incubated for 1 h on ice. After centrifugation at 16,100g for 10 min at 4°C, the supernatants containing the nuclear proteins were collected. Protein concentration was determined using the Bradford assay (Bio-Rad). For AcCoA measurement, non-denatured total and cytosolic fractions were used, and for immunoblot, the fractions were denatured. Histone H3 or GAPDH were used as nuclear or cytosolic control.

Acetyl-Coenzyme A (AcCoA) Measurement

The AcCoA content was determined on total or cytosolic fractions using the PicoProbe AcCoA assay kit (ab87546, Abcam) according to manufacturer's instructions. Briefly, after deproteinization using the perchloric acid, the CoASH Quencher and Quencher remover were added into the sample to correct the background generated by free CoASH and succ-CoA. The sample was then diluted with the reaction mix, and fluorescence was measured using a plate reader and the following settings: λex 535 nm; λem 587 nm. Fluorescence was measured using a Versamax Tunable microplate reader (Molecular Devices). The acetyl-CoA standard curve was made in the range of 0–100 pM and the correlation coefficient was 0.990 or higher.

EP300 Activity Assay

The EP300 activty was determined using the SensoLyte EP300 assay kit (AS-72172, Anaspec) with some modification. Briefly, cell lysates or brain tissue lysates were incubated with AcCoA solution and substrates (H3) for 15 min at 37°C. Developer solution was added and incubated for 30 min at room temperature. The fluorescence was measured using a plate reader and the following settings: λex 389 nm; λem 513 nm. Fluorescence was measured using a Versamax Tunable microplate reader (Molecular Devices).

Co-Immunoprecipitation (Co-IP)

Cells in 60 mm dishes were washed twice with PBS and lysed in ice-cold lysis buffer (40 mM HEPES (pH 7.4), 2 mM EDTA, 10 mM pyrophosphate, 10 mM glycerophosphate, and 0.3% CHAPS (for interaction of mTORC1 with Rag complex) or 0.5% Triton X-100 (for IP with Ac-K antibody) and protease/phosphatase inhibitors cocktail, and further supplemented with 10 mM sodium butyrate and 1 mM TSA for IP with Ac-K antibody. Lysates were incubated on ice for 20 min and isolated by centrifugation at 16,100g for 10 min. Supernatants were transferred to new tubes; 1/10 of the sample was kept as input control, while the remaining lysate was overnight incubated with primary antibodies at 4°C with gentle agitation. Thereafter, Dynabeads-protein G (Life Technologies) were added to the samples and incubated at 4°C for 2 h. Beads were washed three times with lysis buffer and the immunoprecipitated proteins were eluted and denatured with 2 X Laemmli buffer and boiled for 5 min. For co-transfection experiments, HeLa cells were transfected with the indicated plasmids as follows: 1000 ng or 500 ng myc-mTOR in pRK5 or peYFP-C1-mTOR; 500 ng HA-, YFP-Raptor with or without point mutation in pRK5; 100 ng HA-GST-RagB in pRK5; 100 ng HA-GST-RagD in pRK5 or Flag-RagD in pLJM1. The total amount of plasmid DNA in each transfection was normalized to 2 μg with empty pRK5. For analysis of acetylated Raptor in tissues, we used tissue lysis buffer (20 mM Tris-HCl (pH7.4), 5 mM EDTA, 150 mM NaCl, 0.5% Triton X-100, 10 mM sodium butyrate, 1 mM TSA and protease/phosphatase inhibitors cocktail) and 150 μg of lysates was used for IP.

GFP-Trap for IP of GFP (or YFP)-Fusion Proteins

The IP of GFP or YFP-fusion proteins was performed using GFP-Trap (gtma-100, ChromoTek) according to manufacturer's instructions with some modification. Briefly, cells in 60 mm dishes were lysed in ice-cold lysis buffer (10 mM Tris-HCl, pH 7.4, 150 mM NaCl, 0.5 mM EDTA and 0.3% CHAPS and protease/phosphatase inhibitors cocktail). 0.5ml of cell lysate was incubated for 1 h at 4°C with

20 μl of GFP-Trap slurry, and then the beads were washed 2 times with the wash buffer (10 mM Tris-HCl, pH 7.4, 150 mM NaCl, 0.5 mM EDTA). 60 μl of 2X sample buffer were added, and boiled for 10 min at 100°C, separated by SDS-PAGE.

Mitochondrial Morphology Analysis

Mitochondrial morphology was investigated using MitoTracker green FM (M7514, Invitrogen) according to manufacturer's instructions. Briefly, control or MCCC1 siRNA transfected cells for 48 h were stained with 100 nM MitoTracker Green FM for 20 min to visualize mitochondrial morphology and then washed 2 times with Opti-MEM. Images were captured with LSM880 Zeiss confocal with 63X oil-immersion lens and quantified using the ImageJ program. These procedures were performed in a blinded fashion.

Assessment of Oxidative Stress

For analyzing the oxidative stress on control or MCCC1 knockdown cells, cells were incubated in 2 μM of MitoSOX Red reagent (Molecular Probes), which is a specific superoxide marker in mitochondria, and 500 nM of Mitotracker Green FM (Molecular Probes) for 10 min at 37°C. Fluorescent intensity and distribution were observed using LSM880 Zeiss confocal with 63X oil-immersion lense. The intensity was measured using Volocity software.

QUANTIFICATION AND STATISTICAL ANALYSIS

Image Analysis

Volocity software (PerkinElmer) was used for analysis and processing of confocal images. For co-localization analysis of confocal images, we used Mander's Overlap Coefficient (MOC) or Pearson's Correlation Coefficient (PCC). A minimum of 30 cells were examined for each condition. All experiments were repeated at least three times. The background was fixed for all within-experiment analyses. See details in the figure legends.

Statistical Analysis

Significance levels for comparisons between groups were determined with unpaired or paired two-tailed Student's t-test using GraphPad Prism 5 (GraphPad Software) or Excel (Microsoft Office), where appropriate. For western blots, protein levels were normalized to total forms or a housekeeping protein, such as tubulin or GAPDH. All data were expressed as means ± standard error of the mean (S.E.M). P values of < 0.05 were considered statistically significant. Sample sizes were chosen on the basis of extensive experience with the assays we have performed. The experiments were appropriately randomized. We have not formally tested whether the data met the assumptions of the statistical approach, but these assays and data are typically analysed in the literature with the approaches we have used. The statistical parameters are specified within the figure legends.

DATA AND SOFTWARE AVAILABILITY

The authors declare that the data supporting the findings of this study are available within the article and its Supplemental Information files.

Cell Metabolism, Volume *29*

Supplemental Information

Leucine Signals to mTORC1 via Its Metabolite Acetyl-Coenzyme A

Sung Min Son, So Jung Park, Huikyong Lee, Farah Siddiqi, Jong Eun Lee, Fiona M. Menzies, and David C. Rubinsztein

Figure S1.

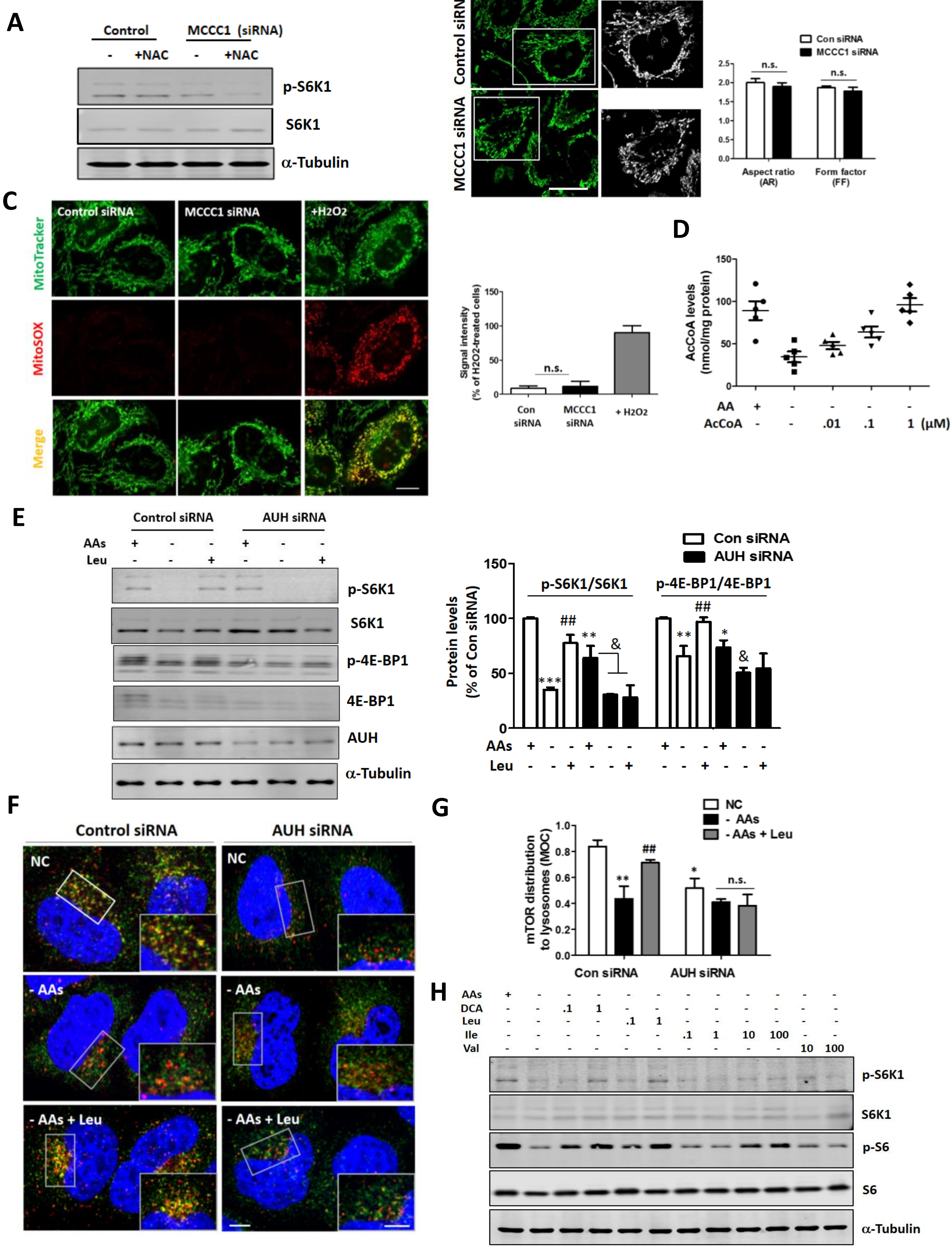
A
Control
MCCC1 (siRNA)
- +NAC - +NAC
p-S6K1
S6K1
α-Tubulin
B
Control siRNA
MCCC1 siRNA
Con siRNA
MCCC1 siRNA
n.s.
n.s.
Aspect ratio (AR)
Form factor (FF)
C
Control siRNA
MCCC1 siRNA
+H2O2
MitoTracker
MitoSOX
Merge
Signal intensity (% of H2O2-treated cells)
n.s.
Con siRNA
MCCC1 siRNA
+ H2O2
D
AcCoA levels (nmol/mg protein)
AA + - - - -
AcCoA - - .01 .1 1 (μM)
E
Control siRNA
AUH siRNA
AAs + - - + - -
Leu - - + - - +
p-S6K1
S6K1
p-4E-BP1
4E-BP1
AUH
α-Tubulin
Con siRNA
AUH siRNA
Protein levels (% of Con siRNA)
p-S6K1/S6K1
p-4E-BP1/4E-BP1
F
Control siRNA
AUH siRNA
NC
- AAs
- AAs + Leu
G
NC
- AAs
- AAs + Leu
mTOR distribution to lysosomes (MOC)
n.s.
Con siRNA
AUH siRNA
H
AAs
DCA
Leu
Ile
Val
p-S6K1
S6K1
p-S6
S6
α-Tubulin

Figure S1. Related to Figure 1.

(A) The role of ROS scavenger N-acetylcysteine (NAC) on mTORC1 regulation in control and MCCC1 knockdown HeLa cells. Blots are representative of at least 3 independent experiments (N=3). p-S6K1 (Thr389).

(B) Mitochondrial morphology in control and MCCC1 knockdown HeLa cells using MitoTracker green dye. Scale bar, 5 μm. Values are mean ± S.E.M. n = about 150 cells. n.s, not significant.

(C) Mitochondrial superoxide levels in control and MCCC1 knockdown HeLa cells. H_2O_2 is a positive control for generation of mitochondrial superoxide. Scale bar, 5 μm. Values are mean ± S.E.M. n = about 100 cells.

(D) AcCoA levels in HeLa cell lysates after AcCoA treatment in the media. We used AcCoA at concentration of 1 μM for this study unless otherwise indicated. N=4.

(E) mTORC1 regulation by Leu under AA starved condition in control and AUH knockdown HeLa cells. * $p < 0.05$, ** $p < 0.01$, *** $p < 0.001$ vs. control cells; ## $p < 0.01$ vs. AA starved cells; & $p < 0.05$ vs. AUH knockdown HeLa cells (two-tailed t-test). NC; normal control. N=3. p-S6K1 (Thr389), p-4E-BP1 (Thr37/46).

(F) Control and AUH knockdown HeLa cells were either left untreated, AA starved for 2 h, or AA starved and then Leu was added for 0.5 h, then immunostained with mTOR (green) and LAMP1 (red) antibodies as shown. Enlarged panels show an overlap between mTOR and LAMP1 signals. The fraction of mTOR-positive lysosomes were determined using Volocity software. Scale bar, 5 μm, 1 μm (enlarged images). NC, normal control.

(G) Quantification data of Figure S1F. Values are mean ± S.E.M. n = about 60 cells. * $p < 0.05$, ** $p < 0.01$ vs. control cells; ## $p < 0.01$ vs. AA starved cells (two-tailed t-test).

(H) mTORC1 regulation by DCA or BCAAs (Leu, Ile and Val). N=3. Unit, μM. p-S6 (Ser235/236).

Figure S2.

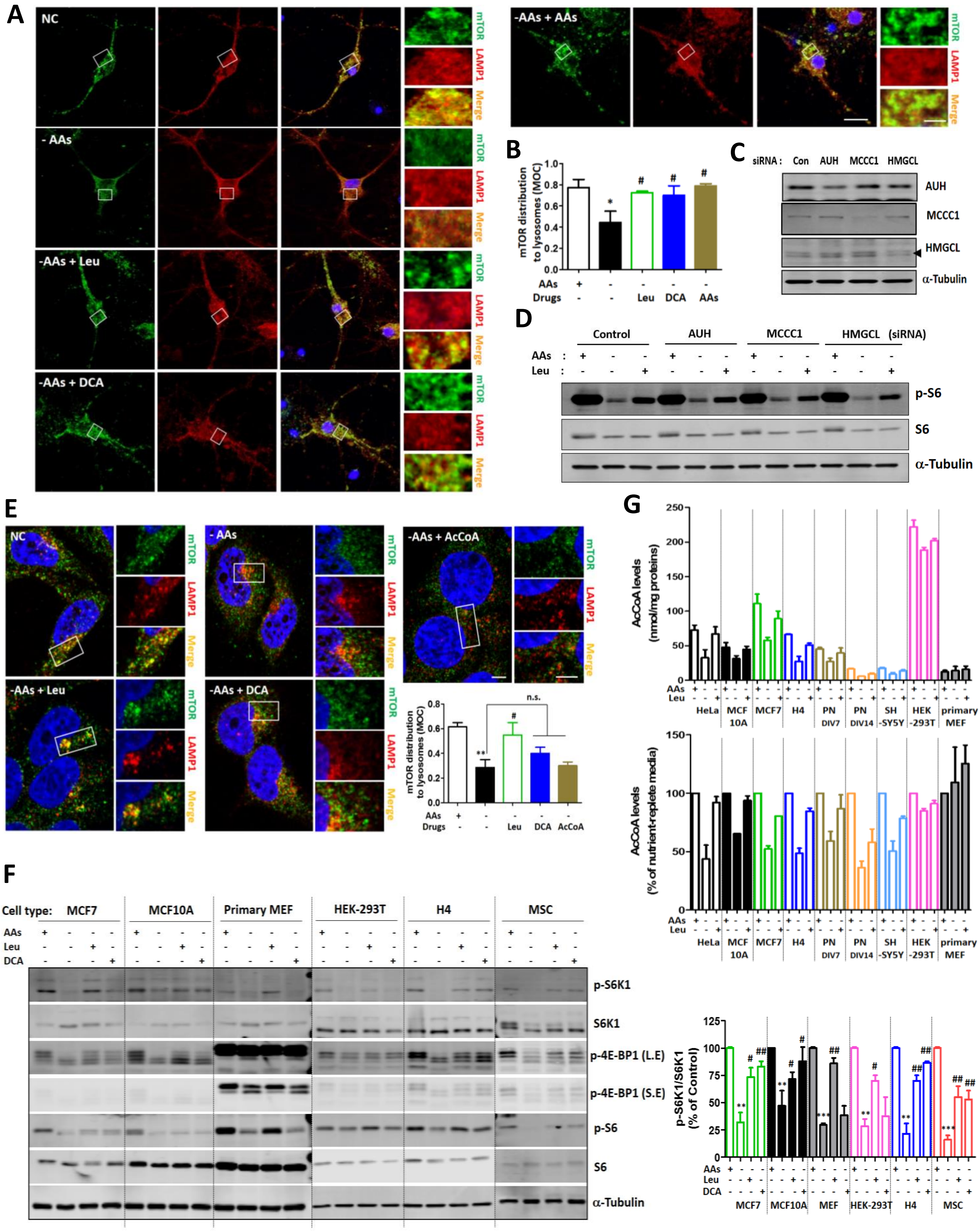
A
NC
- AAs
-AAs + Leu
-AAs + DCA
-AAs + AAs
mTOR
LAMP1
Merge
B
mTOR distribution to lysosomes (MOC)
AAs
Drugs
Leu
DCA
AAs
C
siRNA :
Con
AUH
MCCC1
HMGCL
α-Tubulin
D
Control
AUH
MCCC1
HMGCL (siRNA)
AAs :
Leu :
p-S6
S6
α-Tubulin
E
NC
- AAs
-AAs + AcCoA
-AAs + Leu
-AAs + DCA
n.s.
mTOR distribution to lysosomes (MOC)
AcCoA
F
Cell type:
MCF7
MCF10A
Primary MEF
HEK-293T
H4
MSC
AAs
Leu
DCA
p-S6K1
S6K1
p-4E-BP1 (L.E)
p-4E-BP1 (S.E)
p-S6
S6
α-Tubulin
p-S6K1/S6K1 (% of Control)
MCF7
MCF10A
MEF
HEK-293T
H4
MSC
G
AcCoA levels (nmol/mg proteins)
AcCoA levels (% of nutrient-replete media)
HeLa
MCF 10A
MCF7
H4
PN DIV7
PN DIV14
SH -SY5Y
HEK -293T
primary MEF

Figure S2. Related to Figure 2.

(A) mTORC1 distribution onto lysosomal membrane under AA starvation with or without Leu or DCA in primary neurons. Scale bar, 10 μm, 1 μm (enlarged images). N=4.

(B) Quantification data of Figure S2A. Values are mean ± S.E.M. n = about 40 cells. * $p < 0.05$ vs. control cells; # $p < 0.05$ vs. starved cells (two-tailed t-test).

(C) Efficiency check of siRNAs for AUH, MCCC1 and HMGCL in HEK-293T cells. N=3.

(D) mTORC1 regulation by Leu in the presence or absence of AA, the latter with/without Leu, in control HEK-293T cells or in MCCC1, AUH or HMGCL knockdown cells. N=3.

(E) mTORC1 distribution onto lysosomal membrane under AA starvation with or without Leu, DCA or AcCoA on HEK-293T cells. Values are mean ± S.E.M. n = about 50 cells. ** $p < 0.01$ vs. control cells; # $p < 0.05$ vs. AA starved cells. Scale bar, 2 μm, 0.5 μm (enlarged images).

(F) mTORC1 regulation by Leu or DCA in several cell types. MSC, mesenchymal stem cells. Values are mean ± S.E.M. ** $p < 0.01$, *** $p < 0.001$ vs. control cells; # $p < 0.05$, ## $p < 0.01$ vs. AA starved cells (two-tailed t-test). N=3.

(G) AcCoA levels in several cell types. N=2. PN, primary neurons; DIV, days in vitro. Both actual concentration of AcCoA and % of nutrient-replete media were represented.

Figure S3.

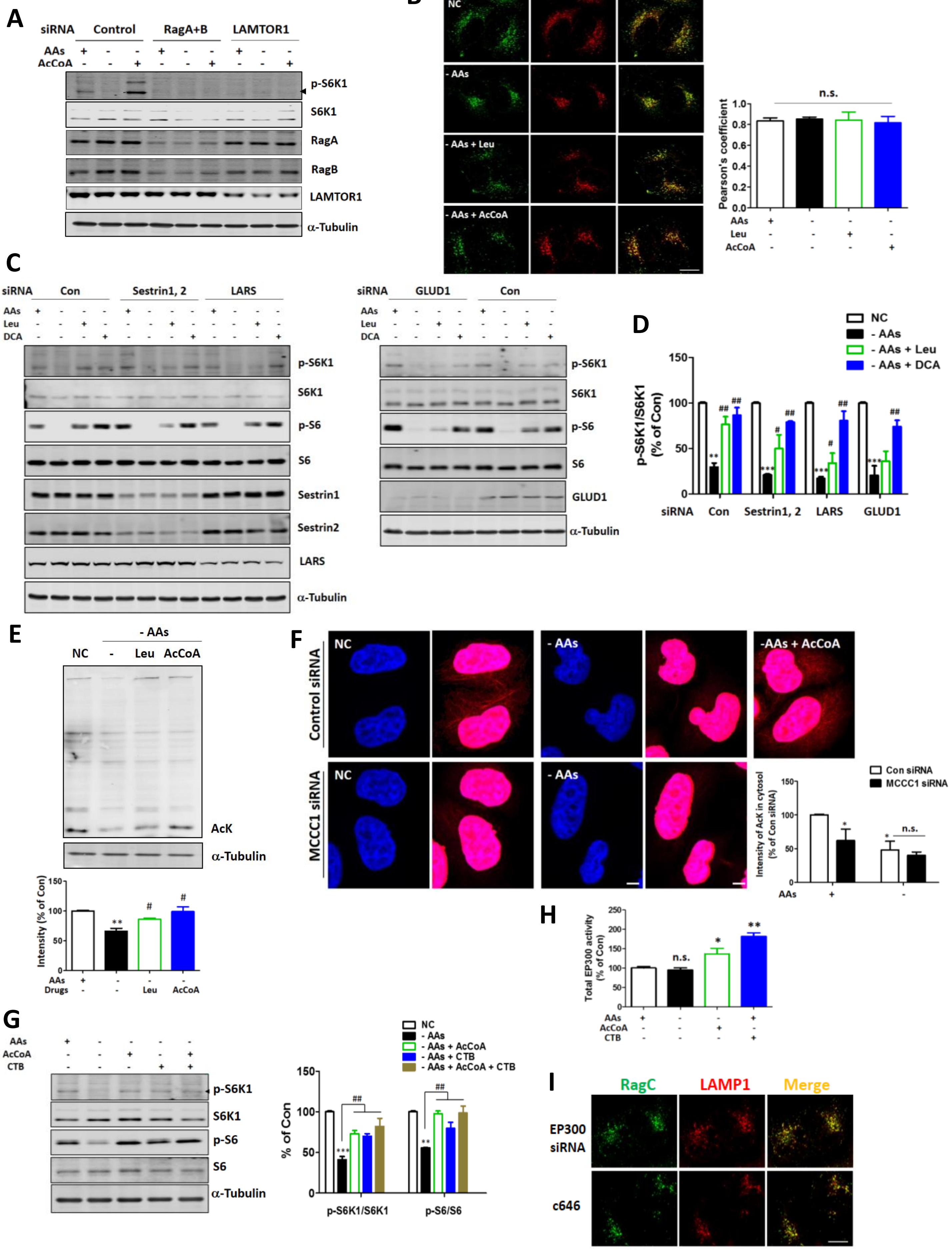
A
siRNA Control RagA+B LAMTOR1
AAs
AcCoA
p-S6K1
S6K1
RagA
RagB
LAMTOR1
α-Tubulin
B
RagC LAMP1 Merge
NC
- AAs
- AAs + Leu
- AAs + AcCoA
Pearson's coefficient
n.s.
AAs
Leu
AcCoA
C
siRNA Con Sestrin1, 2 LARS
AAs
Leu
DCA
p-S6K1
S6K1
p-S6
S6
Sestrin1
Sestrin2
LARS
α-Tubulin
siRNA GLUD1 Con
GLUD1
D
NC
- AAs
- AAs + Leu
- AAs + DCA
p-S6K1/S6K1 (% of Con)
siRNA Con Sestrin1, 2 LARS GLUD1
E
- AAs
NC - Leu AcCoA
AcK
α-Tubulin
Intensity (% of Con)
AAs
Drugs
F
NC
- AAs
-AAs + AcCoA
Control siRNA
MCCC1 siRNA
Con siRNA
MCCC1 siRNA
Intensity of AcK in cytosol (% of Con siRNA)
n.s.
AAs
G
AAs
AcCoA
CTB
p-S6K1
S6K1
p-S6
S6
α-Tubulin
NC
- AAs
- AAs + AcCoA
- AAs + CTB
- AAs + AcCoA + CTB
% of Con
p-S6K1/S6K1
p-S6/S6
H
Total EP300 activity (% of Con)
n.s.
AAs
AcCoA
CTB
I
RagC LAMP1 Merge
EP300 siRNA
c646

Figure S3. Related to Figure 3.

(A) mTORC1 regulation by AcCoA in control, or RagA/B or LAMTOR1 knockdown HeLa cells. Blots are representative of at least 3 independent experiments. (N=3)

(B) Association of RagC on lysosomal membrane under AA starvation with or without Leu or AcCoA. Values are mean ± S.E.M. n = about 50 cells. Scale bar, 5 μm. n.s., not significant.

(C) mTORC1 regulation by Leu or DCA in Sestrin 1 and 2 (Sestrin1, 2), LARS or GLUD1 knockdown HeLa cells. N=4.

(D) Quantification data of Figure S3C. Values are mean ± S.E.M. ** $p < 0.01$, *** $p < 0.001$ vs. control HeLa cells; # $p < 0.05$, ## $p < 0.01$ vs. AA starved HeLa cells (two-tailed t-test).

(E) Change in global acetylation under AA starved conditions in HeLa cells. Representative immunoblots of total cell lysates showing a change in protein acetylation after 2 h of AA starvation, and then with or without adding with Leu or AcCoA for 0.5 h. ** $p < 0.01$ vs. control cells; # $p < 0.05$ vs. AA starved cells. N=3.

(F) Representative immunofluorescence microphotographs reflecting detection of lysine-acetylated proteins after 2 h of AA starvation with or without adding with AcCoA on control and MCCC1-knockdown HeLa cells. n = about 50 cells. Scale bar represents 2 μm. * $p < 0.01$ vs. control cells.

(G) EP300 activator CTB can rescue mTORC1 activity in AAs starved HeLa cells. ** $p < 0.01$, *** $p < 0.001$ vs. control cells; ## $p < 0.01$ vs. AA starved cells. N=3.

(H) EP300 activity from total cell lysates after AA starvation with/without AcCoA or CTB. * $p < 0.05$, ** $p < 0.01$ vs. control cells. N=3.

(I) Association of RagC on lysosomal membrane on EP300 knockdown or EP300 inhibitor treated cells. n = about 50 cells. Scale bar, 5 μm.

Figure S4.

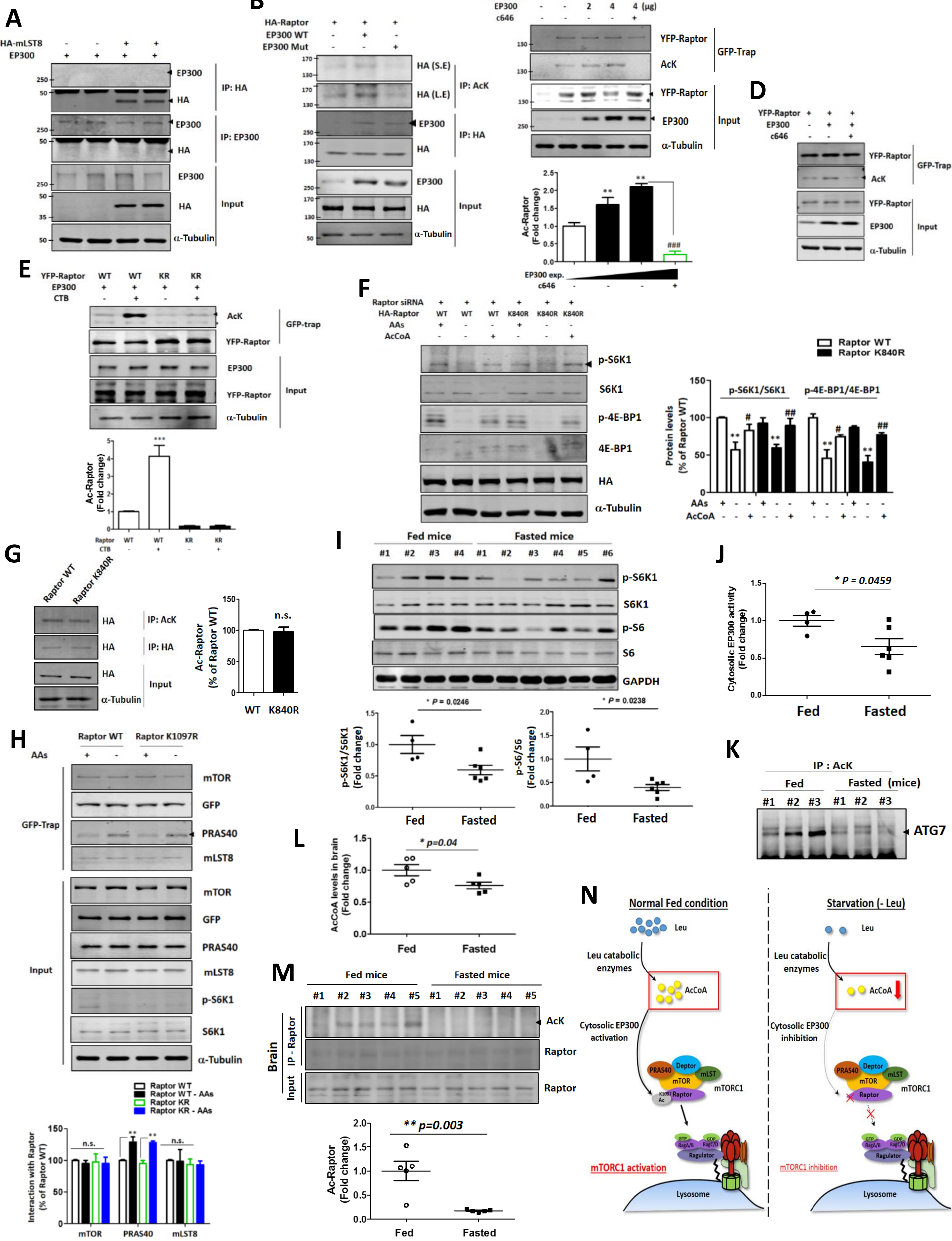
A
HA-mLST8
EP300
IP: HA
IP: EP300
Input
α-Tubulin
B
HA-Raptor
EP300 WT
EP300 Mut
HA (S.E)
HA (L.E)
IP: AcK
IP: HA
C
YFP-Raptor
EP300
c646
GFP-Trap
AcK
Input
Ac-Raptor (Fold change)
EP300 exp.
D
E
CTB
GFP-trap
F
Raptor siRNA
AAs
AcCoA
p-S6K1
S6K1
p-4E-BP1
4E-BP1
Raptor WT
Raptor K840R
p-S6K1/S6K1
p-4E-BP1/4E-BP1
Protein levels (% of Raptor WT)
G
Ac-Raptor (% of Raptor WT)
n.s.
H
Raptor K1097R
mTOR
GFP
PRAS40
mLST8
Interaction with Raptor (% of Raptor WT)
I
Fed mice
Fasted mice
p-S6
S6
GAPDH
* P = 0.0246
* P = 0.0238
J
Cytosolic EP300 activity (Fold change)
* P = 0.0459
Fed
Fasted
K
IP : AcK
Fasted (mice)
ATG7
L
AcCoA levels in brain (Fold change)
* p=0.04
M
Brain
IP - Raptor
** p=0.003
N
Normal Fed condition
Starvation (- Leu)
Leu
Leu catabolic enzymes
AcCoA
Cytosolic EP300 activation
Cytosolic EP300 inhibition
Deptor
mTORC1
Ragulator
Lysosome
mTORC1 activation
mTORC1 inhibition

Figure S4. Related to Figure 4.

(A) mLST8 does not interact with EP300 using IP with HA or EP300 antibody. N=3.

(B) Acetylation of Raptor by EP300 acetyltransferase with EP300 WT and catalytically-inactive EP300 construct (EP300 Mut). EP300 can bind Raptor and acetylate it. S.E., short exposure; L.E., long exposure. N=4.

(C and D) Acetylation of Raptor by EP300 in HeLa (C) and HEK-293T cells (D). EP300 inhibitor c646 inhibits acetylation of Raptor. ** $p < 0.01$ vs. YFP-Raptor expressing cells without transfection of exogenous EP300 cDNA; ### $p < 0.001$ vs. YFP-Raptor and exogenous EP300 expressing cells. N=3.

(E) Acetylation of Raptor on Raptor WT or KR expressing HeLa cells with/without CTB. *** $p < 0.001$ vs. YFP-Raptor expressing cells. N=3.

(F) Cells were depleted of Raptor with siRNA and reconstituted with Raptor wild-type (WT) or K840R mutant, then analysed for mTORC1 activity in the presence or absence of AA, the latter with/without AcCoA in HeLa cells. ** $p < 0.01$ vs. control cells; # $p < 0.05$, ## $p < 0.01$ vs. AA starved cells. N=3.

(G) Acetylation of WT and K840R Raptor. K840 site in Raptor is not acetylated in HeLa cells. N=3. n.s., not significant.

(H) Binding of Raptor WT or K1097R mutant with components of mTORC1. ** $p < 0.01$ vs. control cells. N=3.

(I-K) Decreased mTORC1 (I), EP300 activity (J) and ATG7 acetylation (K) in fasted mouse brains. Brain samples from fed (n=4) and fasted mice (n=6) were analysed. * $p < 0.05$ vs. fed mice.

(L and M) Decreased AcCoA levels and acetylation of Raptor in brains of mice fasted for 23 h. The tissue samples from fed (n=5) and fasted mice (n=5) were analysed for AcCoA (L) and acetylated Raptor (M) levels. * $p < 0.05$, ** $p < 0.01$ vs. fed mice.

(N) Schematic diagram of this study. AA starvation induces decreased AcCoA levels both total and cytosolic fraction that promote the translocation of EP300 into nucleus. Because EP300 acetyltransferase can bind Raptor and acetylate it at K1097 site which is important for interaction with Rag complex, AA starvation induces Raptor deacetylation, and then results in inhibition of interaction with Rag complex on the lysosomal membrane. The knockdown of Leu catabolic enzymes (MCCC1 and AUH) shows similar pattern with AA starvation, suggesting Leu metabolite AcCoA has an essential role on sensing Leu for mTORC1

activation. Interestingly, this mechanism appears to be relevant in a variety of cell types, including HeLa and neurons, not MEF and HEK-293T cells.

Table S1. siRNA sequences, Related to STAR Methods

Non-targeting SMARTpool siRNA<br>UGGUUUACAUGUCGACUAA<br>UGGUUUACAUGUUGUGUGA<br>UGGUUUACAUGUUUUCUGA<br>UGGUUUACAUGUUUUCCUA	Dharmacon	D-001810-10
RagA siRNAa: CCGAUGAAGUUCUGCUGUU	Dharmacon	J-016070-05
RagA siRNAb: GACAAUAUCUUCCGUAACG	Dharmacon	J-016070-06
RagA siRNAc: GAACUCUCCUGACGCCAAA	Dharmacon	J-016070-07
RagA siRNAd: UCGAUAAUCUUCGCCAAUU	Dharmacon	J-016070-08
RagB siRNAa: GAAGAAGAUUUGAGGCGUU	Dharmacon	J-012189-09
RagB siRNAb: GGGACAACAUCUUCCGAAA	Dharmacon	J-012189-10
RagB siRNAc: AGUGUAAAGAGCAGCGUGA	Dharmacon	J-012189-11
RagB siRNAd: CUGAGAAAACGACGGAGAA	Dharmacon	J-012189-12
LAMTOR1 siRNAa: GUUUGUCACCCUCGAUAAA	Dharmacon	J-020916-17
LAMTOR1 siRNAb: AAGUGAGGGUAGAACCUUU	Dharmacon	J-020916-18
LAMTOR1 siRNAc: GGCUUAUACAGUACCCUAA	Dharmacon	J-020916-19
LAMTOR1 siRNAd: UCUCCAGGAUAGCUGCUUA	Dharmacon	J-020916-20
AUH siRNAa: GAGCAGUGAUUAACGAUAU	Dharmacon	J-008457-05
AUH siRNAb: GGAGGUCGAUUUAGUAACA	Dharmacon	J-008457-06
AUH siRNAc: AAAGUACGGACCAUAAUAA	Dharmacon	J-008457-07
AUH siRNAd: GAAGAAGCUUGUUAUGCUC	Dharmacon	J-008457-08
HMGCL siRNAa: AAGCAGGACUCUCUGUUAU	Dharmacon	J-019290-05
HMGCL siRNAb: CGUAUCUACUCCAGUGAAA	Dharmacon	J-019290-06
HMGCL siRNAc: UCAAUGGGCUGCUACGAGA	Dharmacon	J-019290-07
HMGCL siRNAd: UCUGAAAGCUGGAAACUUU	Dharmacon	J-019290-08
KAT2A siRNAa: AGGACAAAUUGGUGCCCGA	Dharmacon	J-008457-17
KAT2A siRNAb: GUUCCUGGCAUUCGAGAGA	Dharmacon	J-008457-18
KAT2A siRNAc: GCUACUACGUGACCCGGAA	Dharmacon	J-008457-19
KAT2A siRNAd: ACUCAUGUCUUUGGGCGAA	Dharmacon	J-008457-20
KAT2B siRNAa: GGUACUACGUGUCUAAGAA	Dharmacon	J-005055-05
KAT2B siRNAb: GAGCCGACCUGCAGCAAAU	Dharmacon	J-005055-06
KAT2B siRNAc: CGACAGAUUCCUAUAGAAA	Dharmacon	J-005055-07
KAT2B siRNAd: GCAAACAAUAGUUGAGUUG	Dharmacon	J-005055-08
KAT5 siRNAa: GCGUCCAUUACAUUGACUU	Dharmacon	J-006301-05
KAT5 siRNAb: GCCUCAAUCUCAUCAACUA	Dharmacon	J-006301-06
KAT5 siRNAc: CUAGGUACCUAGCUUCGAU	Dharmacon	J-006301-07
KAT5 siRNAd: GAUGGUACCAUCAGUGCCC	Dharmacon	J-006301-08
KAT8 siRNAa: GGGAACUACGAAAUUGGGU	Dharmacon	J-014800-05
KAT8 siRNAb: AUCCCUCAAUAGGUACCUA	Dharmacon	J-014800-06
KAT8 siRNAc: CUACGUGGGCUUUAAGGUA	Dharmacon	J-014800-07

KAT8 siRNAd: AUGCCUGGUAUUUGGUACC	Dharmacon	J-014800-08
EP300 siRNAa: GGACUACCCUAUCAAGUAA	Dharmacon	J-003486-11
EP300 siRNAb: GACAAGGGAUAAUGCCUAA	Dharmacon	J-003486-12
EP300 siRNAc: GUUCAAUAAUGCCUGGUUA	Dharmacon	J-003486-13
EP300 siRNAd: CGACAGGGAUGCAGCAACA	Dharmacon	J-003486-14
Raptor siRNAa: UGGCUAGUCUGUUUCGAAA	Dharmacon	J-004107-05
Raptor siRNAb: CACGGAAGAUGUUCGACAA	Dharmacon	J-004107-06
Raptor siRNAc: AGAAGGGCAUUACGAGAUU	Dharmacon	J-004107-07
Raptor siRNAd: UGGAGAAGCGUGUCAGAUA	Dharmacon	J-004107-08
Sestrin1 siRNAa: GAGGAGGAUCAGCGGUUGA	Dharmacon	J-020244-05
Sestrin1 siRNAb: GCUCAAUGGUUUAGAGAAU	Dharmacon	J-020244-06
Sestrin1 siRNAc: CAGCAGAGAUUCAACUACU	Dharmacon	J-020244-07
Sestrin1 siRNAd: UUAGUGAACCUGCAUGUAA	Dharmacon	J-020244-08
Sestrin2 siRNAa: GGAGGGAGUAUUAGAUUAU	Dharmacon	J-019134-17
Sestrin2 siRNAb: GCAGGGACCCGUUGAACAA	Dharmacon	J-019134-18
Sestrin2 siRNAc: GCGCAAACUCAGCGAGAUC	Dharmacon	J-019134-19
Sestrin2 siRNAd: CUUUCGGAUAUGAGGACUU	Dharmacon	J-019134-20
LARS siRNAa: GCAAAGAACUAUCCACCUU	Dharmacon	J-010171-05
LARS siRNAb: GGGAAGCGGUAUACAAUUU	Dharmacon	J-010171-06
LARS siRNAc: UAAGUGGCCUGAAAGGUAA	Dharmacon	J-010171-07
LARS siRNAd: GAAAUAGAGCUGUAUGGUU	Dharmacon	J-010171-08
ACLY siRNAa: GCACGAAGUCACAAUCUUU	Dharmacon	J-004915-05
ACLY siRNAb: CGAGUGAAGUCGAUAAACA	Dharmacon	J-004915-06
ACLY siRNAc: GAGAGCAAUUCGAGAUUAC	Dharmacon	J-004915-07
ACLY siRNAd: CCACUCCUCUGCUCGAUUA	Dharmacon	J-004915-08
MCCC1 siRNAa: CCAUGAAGUACACAACAGC	Ambion	AM51331
MCCC1 siRNAb: CCAAGUCCUUGGUAAUCUU	Ambion	s32399
MCCC1 siRNAc: GAUCCUAGCAAUAACUUCA	Ambion	s32400
MCCC1 siRNAd: GAGUUGCUAGUAAAGCGAA	Ambion	s32401
GLUD1 siRNA: GGGCCUUUUGAACAGUAGU	Ambion	AM16708
